# Genera specific distribution of DEAD-box RNA helicases in cyanobacteria

**DOI:** 10.1099/mgen.0.000517

**Published:** 2021-02-04

**Authors:** Denise S. Whitford, Brendan T. Whitman, George W. Owttrim

**Affiliations:** ^1^​ Department of Biological Sciences, University of Alberta, Edmonton, AB T6G 2E9, Canada

**Keywords:** CrhR, cyanobacteria, DEAD-box RNA helicase, phylogenetic analysis

## Abstract

Although RNA helicases are essentially ubiquitous and perform roles in all stages of RNA metabolism, phylogenetic analysis of the DEAD (Asp-Glu-Ala-Asp)-box RNA helicase family in a single phylum has not been performed. Here, we performed a phylogenetic analysis on DEAD-box helicases from all currently available cyanobacterial genomes, comprising a total of 362 helicase protein sequences from 280 strains. DEAD-box helicases belonging to three distinct clades were observed. Two clades, the CsdA (cold shock DEAD-box A)-like and RhlE (RNA helicase E)-like helicases, cluster with the homologous proteins from *Escherichia coli *. The third clade, the CrhR (cyanobacterial RNA helicase Redox)-like helicases, is unique to cyanobacteria and characterized by a conserved sequence motif in the C-terminal extension. Restricted distribution is observed across cyanobacterial diversity with respect to both helicase type and strain. CrhR-like and CsdA-like helicases essentially never occur together, while RhlE always occurs with either a CrhR-like or CsdA-like helicase. CrhR-like and RhlE-like proteins occurred in filamentous cyanobacteria of the orders *Nostocales *, *Oscillatoriales* and *Synechococcales *. Similarly, CsdA- and RhlE-like proteins are restricted to unicellular cyanobacteria of the genera *Cyanobium* and *Synechococcus *. In addition, the unexpected occurrence of RhlE in two *Synechococcus* strains suggests recent acquisition and evolutionary divergence. This study, therefore, raises physiological and evolutionary questions as to why DEAD-box RNA helicases encoded in cyanobacterial lineages display restricted distributions, suggesting niches that require either CrhR or CsdA RNA helicase activity but not both. Extensive conservation of gene synteny surrounding the previously described *rimO–crhR* operon is also observed, indicating a role in the maintenance of photosynthesis. The analysis provides insights into the evolution, origin and dissemination of sequences within a single gene family to yield divergent functional roles.

## Data Summary

All of the protein sequences corresponding to the RNA helicases used in this study are publicly available in the GenBank database (https://www.ncbi.nlm.nih.gov). *rpoC2* and most 16S rDNA sequences are also available in the GenBank database. Additional 16S rDNA sequences were obtained from the Joint Genome Institute Integrated Microbial Genomes and Microbiomes (JGI IMG/M) database (https://img.jgi.doe.gov/). Accession numbers are provided within the article and the supplementary data.

**Impact Statement**

RNA helicases are enzymes that affect all aspects of RNA metabolism and, thus, are crucial for regulation of gene expression. Here, we applied a phylogenetic approach to analyse DEAD (Asp-Glu-Ala-Asp)-box RNA helicase distribution across all known cyanobacterial genomes. Cyanobacteria perform oxygenic photosynthesis, constituting an extremely diverse and ancient phylum that has dramatic impacts on primary productivity, water quality, and nitrogen, carbon and oxygen cycling worldwide. The analysis indicated that cyanobacteria only possess DEAD-box RNA helicases belonging to three clades, two of which correspond to groups commonly observed in other bacteria. Members of the third clade, the CrhR (cyanobacterial RNA helicase Redox)-like helicases, are only encoded in cyanobacteria, suggesting a unique role related to maintenance of photosynthesis in this phylum. Restricted distribution of the helicase classes is also observed across cyanobacterial diversity with respect to both helicase type and strain, suggesting niche-specific functionality. The phylum-specific clade and restricted distribution of DEAD-box RNA helicases in cyanobacteria provide unique insights into gene evolution and niche-specific functioning.

## Introduction

Regulation of the expression of gene products occurs at many levels. At the post-transcriptional level, modulation of RNA structure can regulate gene expression by altering RNA stability or translation efficiency. In many cases, these RNA structural changes require interactions with proteins, such as chaperones [1, 2] or helicases [3–5]. Monomeric and multimeric helicases are classified into two large families: superfamily 1 (SF1) and superfamily 2 (SF2) [3–5]. RNA helicases generally belong to SF2 and function in all aspects of RNA metabolism, regulating a diverse range of cellular processes by unwinding and, more rarely, annealing RNA duplexes, performing local strand separation, and by mediating RNA-protein interactions [4, 6–8]. Generally, RNA helicases share a conserved core structure, with two RecA-like functional domains containing a series of conserved sequence motifs that participate in RNA binding, and ATP binding and hydrolysis (Fig. S1, available with the online version of this article) [9–11]. The largest family of RNA helicases, the DEAD (Asp-Glu-Ala-Asp)-box proteins, have 12 highly conserved sequence motifs [7], conferring ATP-dependent local RNA strand separation [12]. Some DEAD-box proteins can also displace proteins from RNA, or clamp to RNA and serve as a nucleation site for the recruitment of other proteins to form functional complexes [6, 7, 13]. In eukaryotes, DEAD-box helicases are essential for survival, as they are intimately involved in crucial cellular processes, including translation, splicing and RNA degradation [7]. Prokaryotic DEAD-box proteins also function in these processes; however, their function is generally only required under non-optimal conditions [14].

Classification of prokaryotic DEAD-box helicases was originally based on the RNA processes in which the protein participates [15, 16], but phylogenetic methods have also been used [14, 17]. Based on conserved sequence domains, López-Ramírez *et al.* [17] classified bacterial DEAD-box proteins into three major groups: (i) proteins that contain a DbpA (DEAD-box protein A) RNA binding domain, including CsdA (cold shock DEAD-box A) and DbpA from *Escherichia coli *, and DeaD/YxiN from *Bacillus subtilis *; (ii) proteins lacking the DbpA domain, including SrmB and RhlB from *E. coli *, and CshA, CshB and YfmL from *B. subtilis *; and (iii) RhlE (RNA helicase E)-like proteins. These classifications, based on sequence and structure, generally relate well to the cellular functions of the DEAD-box protein. For example, the DbpA RNA binding domain facilitates recognition and binding of hairpin 92 of the 23S rRNA by a DEAD-box RNA helicase [18–20]. All of the well-characterized DEAD-box proteins containing this domain, DbpA, CsdA and YxiN, participate in maturation of the 50S ribosomal subunit, and specifically the 23S rRNA [21–23]. These phylogenetic classifications aid in deducing putative functions of DEAD-box helicases from protein sequences as they are annotated in newly sequenced genomes.

*Cyanobacteria* is an ancient phylum of Gram-negative bacteria that evolved as early as 3.5 billion years ago [24]. These bacteria exhibit extreme genetic diversity and are distinguished by being the only known bacteria that perform oxygenic photosynthesis from which they obtain all of their energy [25, 26]. As a group, they have dramatic impacts on cycling of carbon dioxide, fixed carbon, oxygen and nitrogen, and primary productivity on a global scale [27]. In addition, their ability to sequester carbon dioxide makes them ideal platforms for carbon-neutral biotechnological applications [28]. Although phylogenetic analysis of bacterial DEAD-box RNA helicases has been performed [14, 17], extensive analysis within a specific phylum has not been conducted. An initial phylogenetic analysis of cyanobacterial DEAD-box helicases was performed [29]; however, there are a significantly larger number of cyanobacterial genomes now available that makes the analysis more robust. Therefore, it was of interest to evaluate how cyanobacterial DEAD-box RNA helicases relate to those encoded in other bacterial phyla.

Only four DEAD-box RNA helicases have previously been experimentally identified in cyanobacteria: CrhA from *Anabaena variabilis* UTCC 387 [30], CrhB and CrhC from *Nostoc* sp. PCC 7120 [31], and CrhR (cyanobacterial RNA helicase Redox) from *Synechocystis* sp. PCC 6803 (from here on *Synechocystis *) [32], with two examples, CrhA and CrhB, receiving only limited study. CrhC is specifically induced at low temperature [31], and interacts with ribosomes and localizes to the cytoplasmic face of the plasma membrane near the cell poles [33]. Expression of CrhR is regulated in response to the redox poise of the plastoquinone pool of the photosynthetic electron transport chain [32, 34]. CrhR was shown to co-sediment with polysomes and RNA degradosome components, and localize to the thylakoid membrane [35]. Consistent with localization to the thylakoid membrane, *crhR* mutation results in disruption of photosynthesis, particularly at low temperatures [36, 37]. As the cyanobacterial DEAD-box proteins, and in particular CrhR, demonstrate localization and functions unique to cyanobacteria, further study of this protein family across the diversity of cyanobacteria was warranted.

Here, we report a phylogenetic analysis of cyanobacterial DEAD-box proteins indicating that they form three major clades, two with homology to the *E. coli* DEAD-box proteins CsdA and RhlE, and a third unique to cyanobacteria, the CrhR-like helicases. The CrhR-like proteins are found throughout cyanobacteria diversity and share a conserved sequence domain in the C-terminal extension that identifies them as a separate clade within the DEAD-box RNA helicase family. In addition, synteny surrounding the dicistronic *rimO–crhR* operon is extensively conserved, suggesting functional conservation and early acquisition of CrhR-like proteins in the cyanobacterial lineage. The three RNA helicase clades are also encoded in specifically restricted patterns throughout the phylum *Cyanobacteria *. CsdA-like helicases, which occur only in *Synechococcales *, have a DbpA RNA binding motif in the C-terminus and may have been acquired following a loss of the CrhR-like helicase in the ancestral lineage. Strains with RhlE-like helicases also encode at least one other DEAD-box protein in the genome. Based on similarities to characterized homologues, functions for CsdA, CrhR and RhlE in ribosome biogenesis, expression and maintenance of photosynthetic systems, and coordination of CsdA-CrhR function, respectively, are proposed.

## Methods

### Sequence data

Predicted and known amino acid sequences of cyanobacterial DEAD-box RNA helicases were obtained from the National Center for Biotechnology Information (NCBI) non-redundant (nr) protein database. The helicase core domain (amino acids F9–R338) of the sole DEAD-box RNA helicase in *Synechocystis *, CrhR, was retrieved from the NCBI database (accession no. WP_010873784.1) for use as the query sequence. A blastp search, restricted to cyanobacteria (taxid: 1117), yielded 461 unique DEAD-box RNA helicase sequences. Following alignment in mega7, these sequences were manually curated for full-length sequences that contained the 12 conserved motifs of the DEAD-box helicase protein family (Fig. S1). Curation was performed by initially removing sequences that lacked a start or stop codon, and subsequent deletion of sequences that did not span the full length of the helicase core region, extending from F9 to R338 of the *Synechocystis* sp. PCC 6803 *slr0083*-encoded helicase, CrhR. The remaining sequences were aligned with muscle [38] and those containing motifs in the helicase core characteristic of all SF1 subfamily members or other SF2 helicase clades, other than DEAD-box family members, were removed. Following curation, 362 DEAD-box RNA helicase sequences from 280 strains of cyanobacteria were identified. The sequences of the five DEAD-box proteins from *E. coli* were also retrieved from the NCBI to verify branching and clade assignment.

### Sequence alignments and phylogenetic analysis

The helicase sequences were assembled in mega7 v7.0.21 [39], where multiple sequence alignments of the protein sequences were generated using the integrated muscle algorithm [38], using default parameters. This alignment was exported in fasta format and converted to relaxed phylip format using NCLconverter v2.1 [40], accessed via the CIPRES Science Gateway [41].

The maximum-likelihood tree was created using RAxML-HPC Blackbox v8.2.10 [42], accessed via CIPRES [41] using default settings, with the exception of the amino acid substitution matrix, which was set to LG [43]. Rapid bootstrapping ran for 252 replicates before auto-termination with MRE-based bootstrapping criteria [44]. The output tree was visualized in iTOL v4.3.2 [45]. Taxa colours correspond to the cyanobacterial order.

The Euler diagram representing the distribution of the helicase families across cyanobacterial strains was created as a three-set Euler diagram in EulerAPE v.3.0 [46], consisting of CrhR-like, CsdA-like and RhlE-like helicases. The fourth set, for the unclassified helicases, was added with approximate scaling in Adobe Illustrator to visually demonstrate the intersection of the unclassified helicases with the CrhR-like and RhlE-like proteins.

### Gene context analysis

Gene synteny surrounding each DEAD-box protein in the respective cyanobacterial genome was identified using the NCBI Genome Viewer. Related genes were identified from annotations of conserved motifs.

### C-terminal motif identification

Alignments of the C-terminal domains for sequences from each helicase subfamily were generated in muscle [38]. Alignments of a selection of sequences from the subfamily were used for visualization of the alignments. Alignments including all sequences in the subfamily were used to generate sequence logos in WebLogo 3 [47].

### Pfam domain identification

Protein sequences were searched against the Pfam database [48] using the EMBL-EBI web portal with an *E* value cut-off of 1.0.

### *rpoC2* neighbour-joining tree

*rpoC2* gene sequences were identified by searching the genome sequences with the RpoC2 protein of *Synechocystis* (BA000022.2 : 853497–857450). *rpoC2* nucleotide sequences were used instead of 16S rDNA for tree reconstruction as the genome for *Phormidesmis priestleyi* Ana is incomplete, lacking sequences for the 16S rDNA and several core conserved genes often used in concatenated gene trees. *rpoC2* nucleotide sequences were aligned by muscle [38] with default parameters. A neighbour-joining tree was generated in mega7 v7.0.21 [39] and a maximum-likelihood tree was generated using RAxML-HPC Blackbox v8.2.10 [42] using default settings, accessed via the CIPRES [41] Science Gateway.

## Results

### Cyanobacterial DEAD-box RNA helicases form three distinct clades

The amino acid sequence of the *Synechocystis* DEAD-box protein, CrhR, was used to generate a query sequence. The query sequence (F9–R338) was selected as it contained amino acids corresponding to the RNA helicase core, between the conserved phenylalanine upstream of the Q motif [49] and the final arginine of motif VI, HRIGR [50] (Fig. S1), which is highly conserved in all DEAD-box RNA helicases [7]. This sequence was used to search the NCBI non-redundant (nr) protein database, returning 461 unique cyanobacterial records, which were further curated to remove sequences from other SF1 and SF2 helicase families, as well as incomplete sequences. The resulting 362 cyanobacterial DEAD-box protein sequences (Table S1) were aligned, and the phylogenetic relationships were identified by reconstruction of an unrooted maximum-likelihood tree using RAxML [42].

**Fig. 1.**
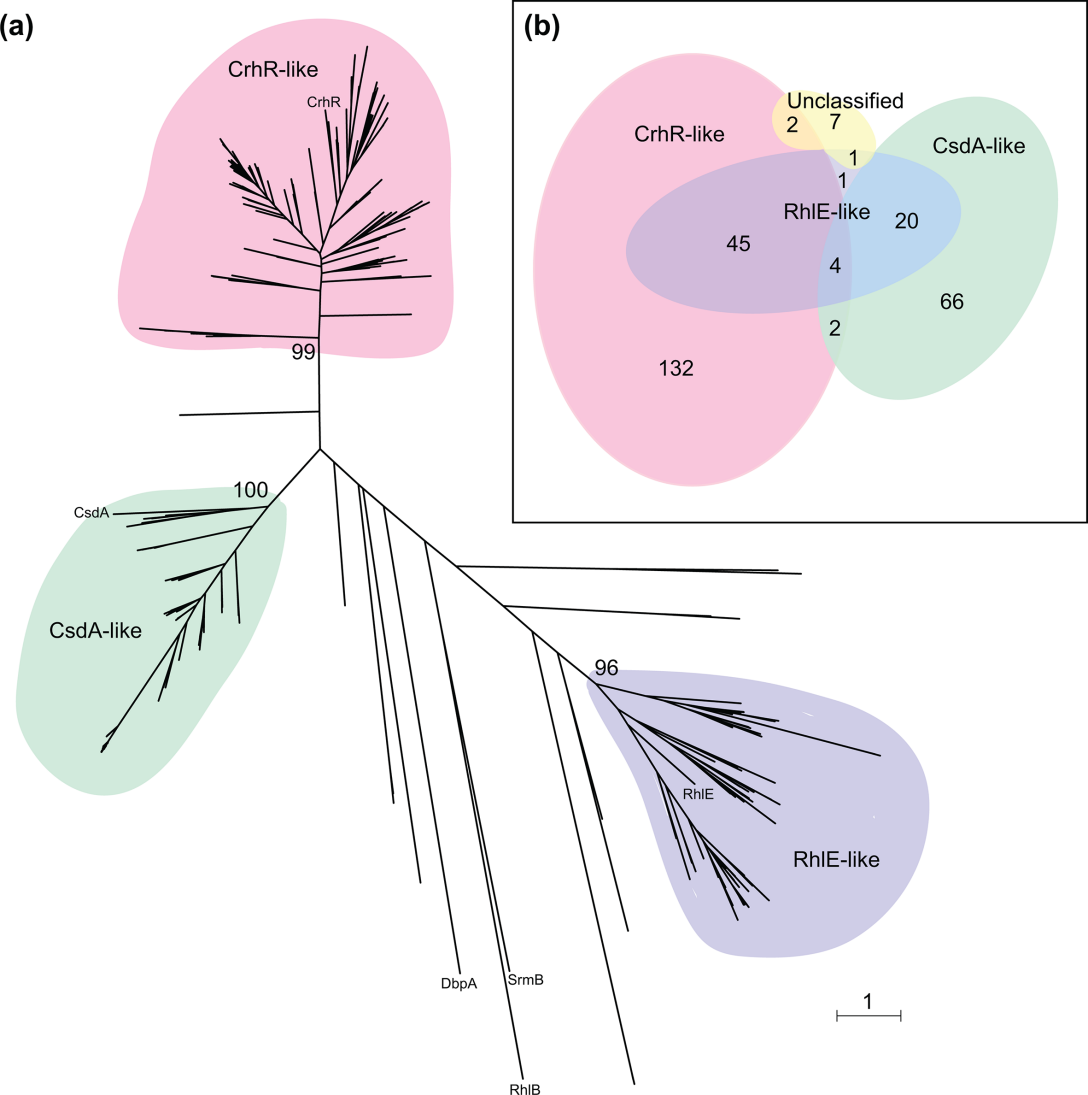
Cyanobacterial DEAD-box proteins cluster into three clades. The phylogenetic tree (a) shows the 362 cyanobacterial DEAD-box proteins form three major clades with bootstrap values ≥95 %, corresponding to CsdA-like and RhlE-like proteins, and a clade unique to cyanobacteria, CrhR-like proteins, named for the sole DEAD-box RNA helicase encoded in the genome of *Synechocystis* sp. PCC 6803. Bootstrap values at the root of each clade, as well as the positions of the five DEAD-box proteins of *E. coli* and CrhR from *Synechocystis* sp. PCC 6803, are indicated. Branch lengths are proportional to the mean number of substitutions per amino acid site as indicated by the scale bar. The distribution of the DEAD-box protein clades identified in the 280 cyanobacterial strains used in the analysis is represented by the Euler diagram shown in the inset (b).

The majority of the cyanobacterial DEAD-box RNA helicase proteins cluster into three clades, each having a bootstrap value ≥95 % (Fig. 1a, b). Two of the *E. coli* helicase sequences, RhlE and CsdA, fall within these groups, while the remaining three, DbpA, RhlB and SrmB, do not group with the cyanobacterial helicase sequences. The three clades were designated as CsdA-like and RhlE-like, corresponding to the *E. coli* helicases that cluster with them, and CrhR-like, for the most-characterized protein sequence in this clade, CrhR, from *Synechocystis* [32]. Of the 362 cyanobacterial DEAD-box proteins in this analysis, 185 fall within the CrhR-like helicases, 92 in CsdA-like and 71 in RhlE-like (Fig. 1b). Fourteen of the cyanobacterial helicase proteins do not cluster with any of the three cyanobacterial clades nor with the three additional DEAD-box RNA helicase subfamilies present in *E. coli* (Fig. 1b).

Examination of helicase distribution demonstrates that 199 cyanobacterial strains contain a single member of one of the DEAD-box clades, 67 contain two forms, while only 4 strains contain members of all three clades (Fig. 1a). Of the strains encoding ungrouped DEAD-box proteins, three strains also encode either a CrhR-like or RhlE-like protein, while the remaining seven encode solely DEAD-box proteins that do not cluster with the three protein subfamilies defined in this study. It is important to note that only a few examples of cyanobacterial strains that do not encode DEAD-box RNA helicases were identified. Interestingly, these include the model organism *Synechococcus elongatus *, of which there are several genome sequences available [51]. DEAD-box proteins were also not identified in some early branching cyanobacteria, including *Synechococcus* sp. JA3-3Ab, *Synechococcus* sp. JA2-3B′a, and the proposed order *Gloeomargaritales *, for which only a single genome exists [52]. Based on the currently available sequence data, it can be concluded that cyanobacterial species encode between zero and three DEAD-box proteins that group into separate clades.

### CrhR-like proteins are conserved across cyanobacterial diversity

The maximum-likelihood tree of the cyanobacterial DEAD-box RNA helicases was also visualized, with the species, strain and order of the cyanobacteria encoding each protein indicated (Fig. S2). CrhR-like proteins were detected in every order of cyanobacteria that encode DEAD-box proteins except *Gloeobacterales* (Fig. S2), the oldest branching extant group of cyanobacteria [52]. This order represents a unique branch of cyanobacteria that lack thylakoid membranes, with photosynthetic light harvesting and electron transport located in the cytoplasmic membrane [53]. Interestingly, while *Gloeobacter violaceus* PCC 7421 and *Gloeobacter kilaueensis* JS1 encode DEAD-box proteins, only one of the *Gloeobacter* proteins clusters weakly with any of the three cyanobacterial DEAD-box helicase subfamilies defined in this study. In addition, CrhR-like helicases were not observed in the available sequences of the phylogenetically related non-photosynthetic proposed classes *Melainabacteria* and *Sericytochromatia* [26]. Conservation of the CrhR-like helicases throughout the diversity of cyanobacteria that evolved following branching from the *Gloeobacterales* and *Gloeomargaritales *, and absence of these proteins from organisms other than cyanobacteria, indicates that it is likely that the ancestral CrhR-like protein arose early in the radiation of the cyanobacterial crown group, after evolution of the non-photosynthetic *Melainabacteria* and the more basal *Sericytochromatia *.

**Fig. 2.**
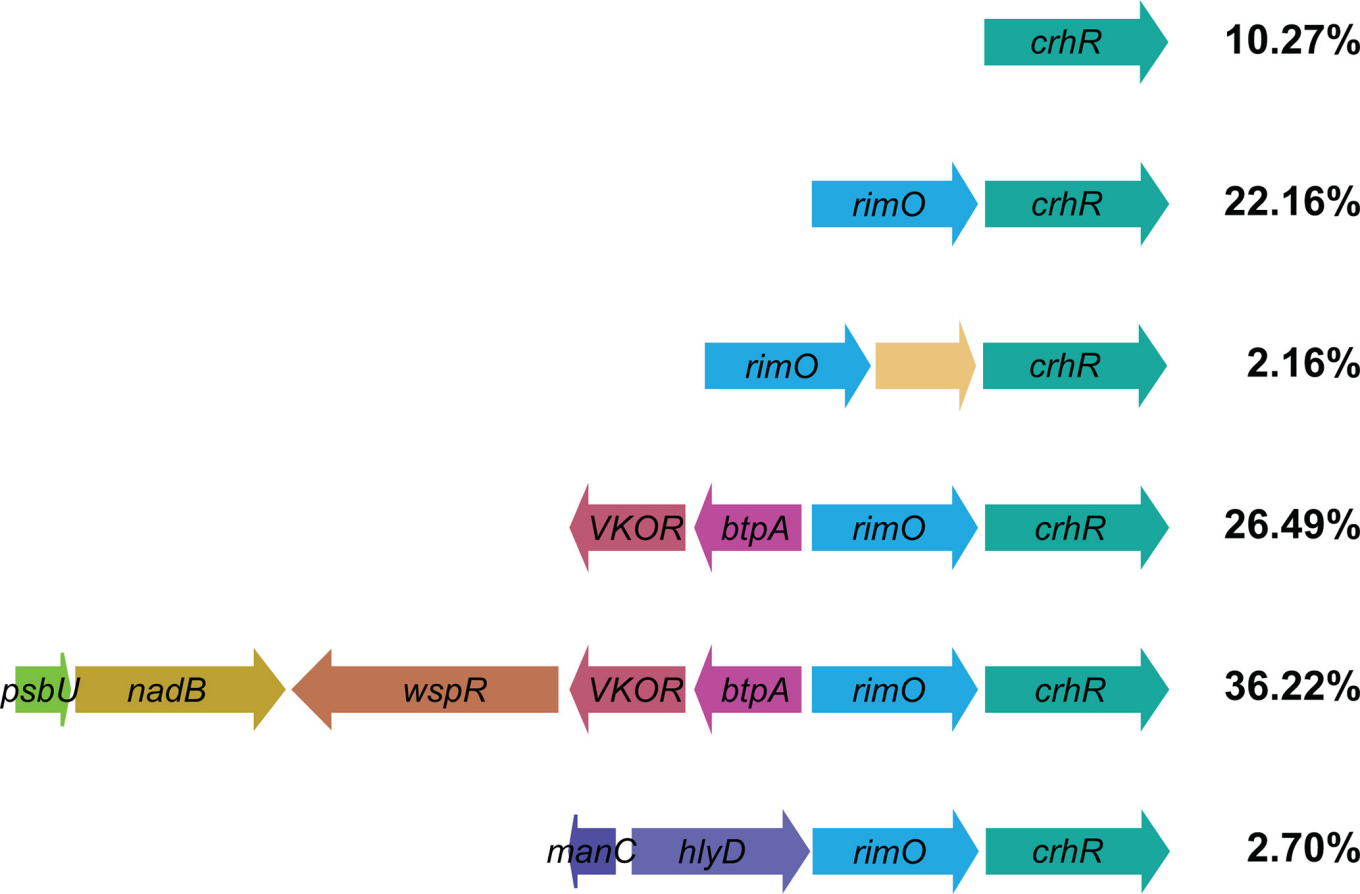
Conserved gene sequence surrounding CrhR-like DEAD-box RNA helicases. Gene synteny surrounding the DEAD-box RNA helicase in strains containing CrhR-like proteins. Percentages represent the relative proportion of the 185 CrhR-encoding cyanobacterial strains in this analysis possessing the indicated gene sequence surrounding CrhR. Functionally related genes have similar colours. The cut-off to be considered as a separate example of conserved gene context was conservation in 2 % of the strains.

Conservation of the CrhR-like helicases in cyanobacteria was also investigated by examination of gene clustering surrounding the helicase. In *Synechocystis *, *crhR* is expressed as a dicistronic operon, downstream of the gene encoding the ribosomal protein S12 methylthiotransferase, *rimO* [54]. Of the 185 cyanobacterial strains that encode CrhR-like helicases in this study, only 19 (10.27 %) do not preserve the synteny of the *rimO–crhR* operon from *Synechocystis* (Fig. 2). Synteny is extensively maintained outside of the *r*i*mO–crhR* operon with 62.71 % of strains encoding a vitamin K epoxide reductase (VKOR) and photosystem I (PSI) stabilizing protein (*btpA*) upstream of the *rimO–crhR* operon (Fig. 2). Of those, 57.7 6% also encode a diguanylate cyclase (*wspR*), l-aspartate oxidase (*nadB*) and photosystem II (PSII) complex extrinsic protein (*psbU*) further upstream. These proteins perform roles in photosynthesis, membrane protein stability, protein folding and cellular energetics. The strong conservation of the *rimO–crhR* gene pair, as well as upstream genes in a preponderance of strains, supports an early acquisition of the CrhR-like proteins in cyanobacteria. Maintenance of the syntenic gene organization throughout cyanobacterial diversity also intimates that the pathways in which the encoded proteins function, including RimO and CrhR, are likely related, as products of highly conserved gene pairs are typically functionally related and often physically interact [55].

### Restricted distribution of DEAD-box proteins in cyanobacterial strains

Strict boundaries for DEAD-box RNA helicase distribution in cyanobacteria was evidenced by genomes that encode both CrhR- and RhlE-like proteins, which are restricted to filamentous cyanobacteria of the orders *Nostocales *, *Oscillatoriales* and *Synechococcales.* Similarly, co-occurrence of CsdA- and RhlE-like proteins is restricted to unicellular cyanobacteria of the genera *Cyanobium* and *Synechococcus *. The three exceptions are *Synechocystis* sp. PCC 7509, *Synechococcus* sp. NIES-970 and *Synechococcus* sp. NKBG15041c, which are unicellular strains that encode both a CrhR-like and RhlE-like helicase. Polyphyly has long been a known issue in the genera *Synechococcus* [56] and *Synechocystis* [57]. 16S rDNA and concatenated gene trees group *Synechocystis* sp. PCC 7509 near strains from the order *Nostocales* rather than other *Synechococcales* [58], suggesting this unicellular cyanobacterium encodes both a CrhR-like and RhlE-like helicase due to its common lineage with *Nostocales* and should be reclassified. In contrast, the other two unicellular strains that encode CrhR and RhlE, *Synechococcus* sp. NIES-970 and *Synechococcus* sp. NKBG15041c, are closely related to *Synechococcus* sp. PCC 7002 [59, 60], which encodes only a CrhR-like helicase. In a species evolution context, the presence of RhlE in only these two strains of unicellular *Synechococcus* is an important observation, since other closely related *Synechococcus* strains only contain CrhR-like helicases. This unpredicted occurrence implies that RhlE was acquired relatively recently in these two strains. Further study will be required to understand the physiological and evolutionary significance of RhlE helicase acquisition by these *Synechococcus* strains.

Furthermore, proteins belonging to the CsdA-like subfamily are only encoded in one cyanobacterial order, primarily in unicellular strains of the *Synechococcales *. This holds for all species examined except for the *Synechococcales* strains *Leptolyngbya* sp. Heron Island J, *Leptolyngbya* sp. PCC 7375 and *Phormidesmis priestleyi* Ana. These three filamentous strains, as well as the unicellular *Synechococcus* sp. PCC 7335, are the only strains that encode DEAD-box proteins from all three subfamilies and are discussed below. Other than the four strains encoding proteins from all three groups, CsdA-like proteins are only encoded in species of *Acaryochloris *, *Cyanobium *, *Prochlorococcus* and *Synechococcus *. The strains from the genera *Cyanobium *, *Prochlorococcus* and *Synechococcus* that encode CsdA are monophyletic, based on both 16S rRNA [60] and concatenated gene [58] phylogenies. The most closely related species of cyanobacteria to these strains is *Synechococcus elongatus* [58], which does not encode a DEAD-box protein.

Co-occurrence of CrhR-like and CsdA-like proteins, excepting the four strains that encode all three subfamilies, is restricted to the genus *Acaryochloris* (Fig. S2). Cyanobacteria belonging to *Acaryochloris *, like *Gloeocapsa *, have divergent photosynthetic pathways, as they are capable of photosynthesis using the far-red spectrum, with chlorophyll *d* as the primary photosynthetic pigment and an altered complement of phycobiliproteins that do not form phycobilisomes on the surface of the thylakoid membrane [61]. As *Acaryochloris* sp. and the four strains that encode all three subfamilies are more distantly related to the other *Cyanobium *, *Prochlorococcus* and *Synechococcus* strains that encode CsdA [58], further analysis will be required to discern whether the CsdA-like proteins were acquired independently by the different cyanobacterial lineages.

### Conserved sequence motif in the C-terminus of CrhR-like proteins characterizes the CrhR-like clade

**Fig. 3.**
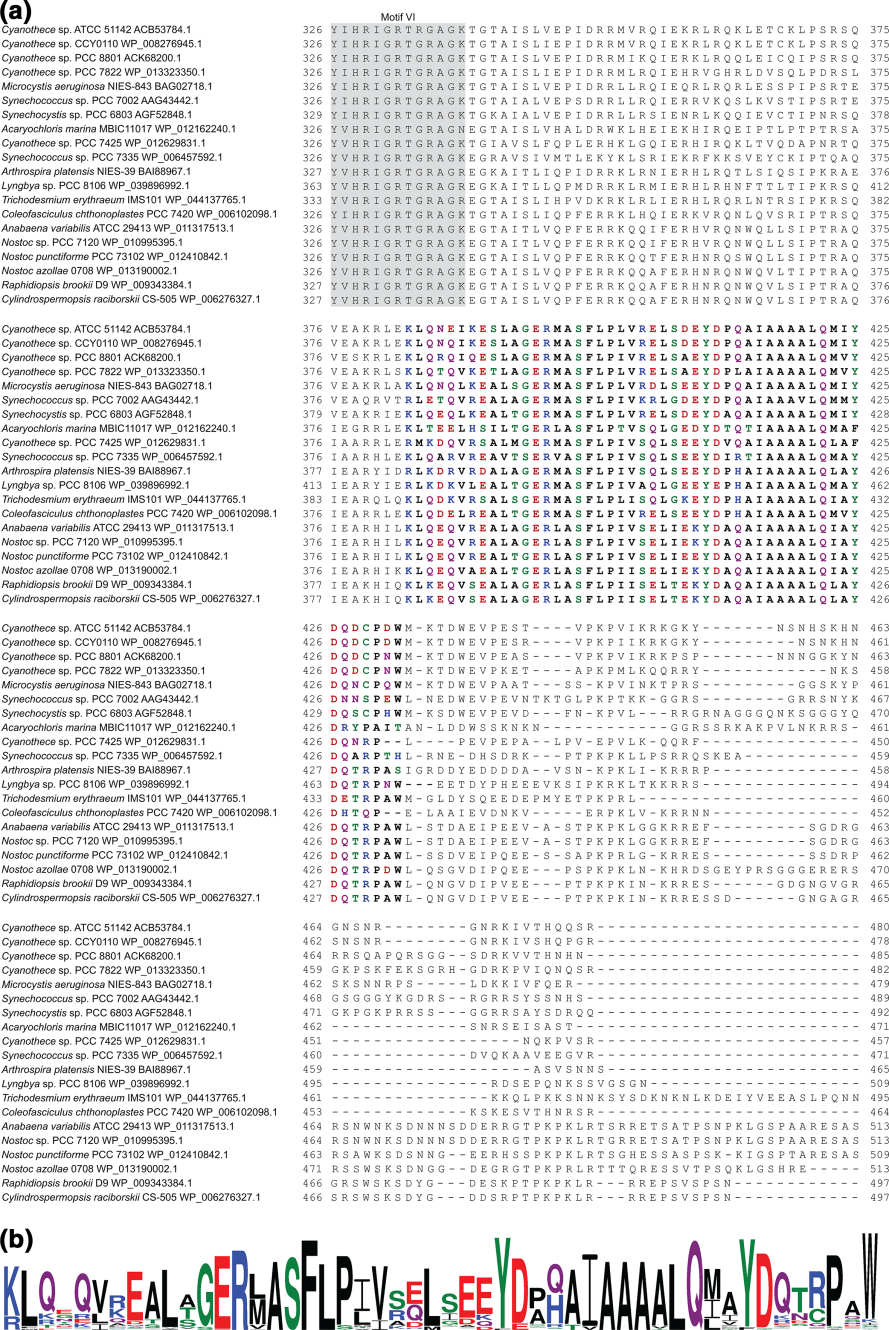
The C-terminal extension of CrhR-like helicases contains a unique, highly conserved sequence motif. (**a**) Alignment of the C-terminal extensions of selected CrhR-like DEAD-box proteins generated by muscle. The final conserved motif in the DEAD-box helicase core, motif VI, HRIGRXXR, is indicated with a grey background. The unique conserved sequence motif characteristic of CrhR-like helicases is indicated in bold, with amino acids coloured based on their chemical properties. (**b**) Sequence logo of the conserved CrhR-specific sequence motif, with the size of the amino acid indicating its conservation. The sequence logo was generated from an alignment of all 185 CrhR-like DEAD-box RNA helicase sequences using WebLogo 3 [47].

**Fig. 4.**
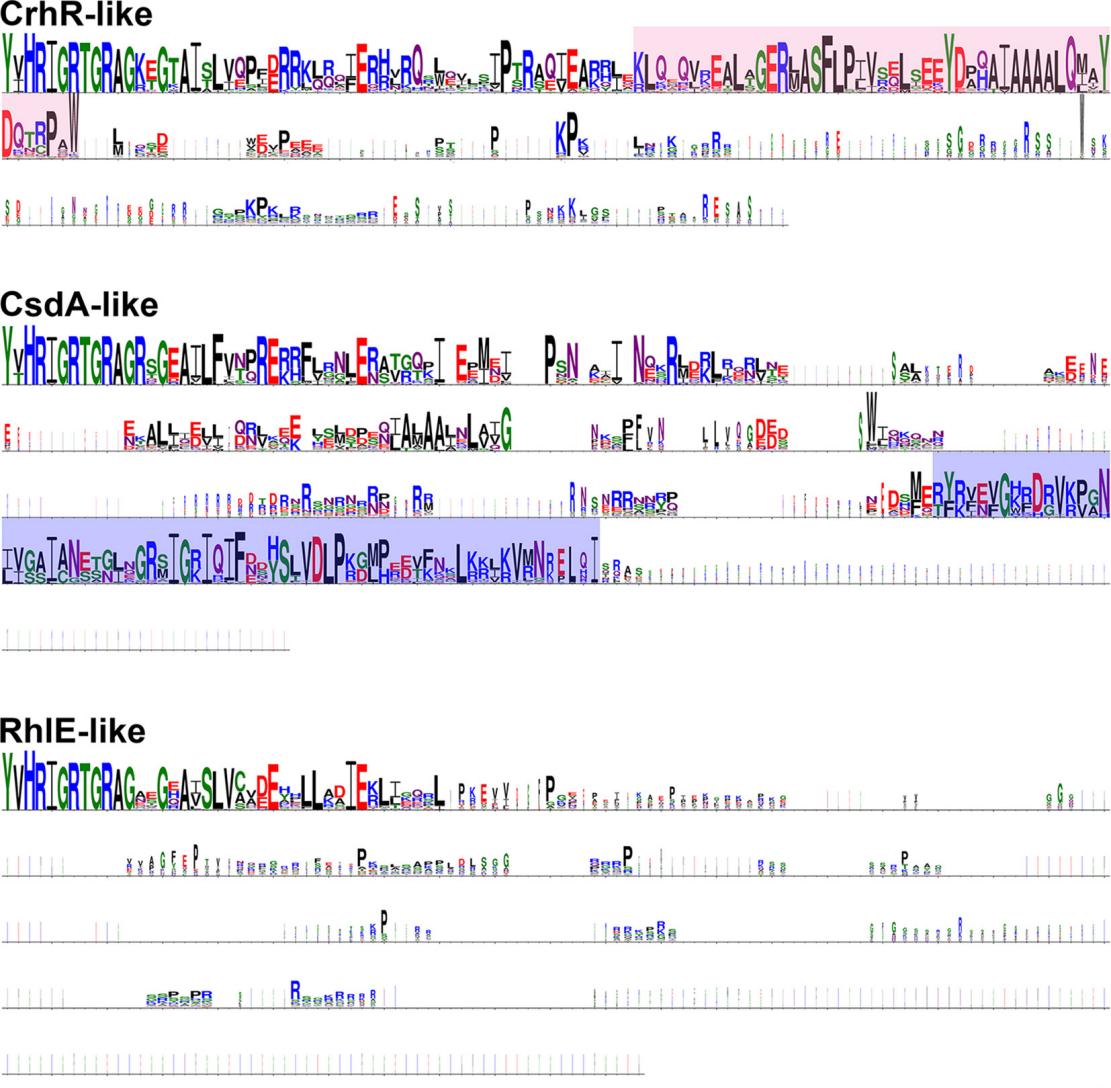
Conservation in the C-terminal extension of cyanobacterial DEAD-box proteins. Comparisons of the conservation in the C-terminal extensions of the three clades of cyanobacterial DEAD-box RNA helicases are shown using sequence logos, with the size of the amino acid indicating its conservation. The CrhR-specific sequence motif is indicated in pink and the DbpA RNA binding domain is marked in blue. Sequence logos were generated in WebLogo 3 from alignments of all cyanobacterial DEAD-box proteins in each clade.

The C-terminal extension in CrhR-like helicases extends 150–200 amino acids downstream of the conserved helicase motif VI, HRIGR (Fig. S1). Within the C-terminal extension, a conserved sequence motif of approximately 50 amino acids is present in all CrhR-like helicases (Fig. 3). This motif is unique to and characterizes the CrhR-like RNA helicase clade as it is not found in the other two clades present in cyanobacteria (Fig. 4) and since other sequences containing this motif were not identified in either the Pfam protein families [48] or the NCBI protein databases. The DEAD-box protein from *G. violaceus* PCC 7421 (WP_011142503.1), the nearest branching protein to the CrhR-like group of DEAD-box proteins, also does not possess the conserved CrhR sequence motif in its C-terminus; however, it does have shorter regions of sequence conservation with the CrhR-like proteins in the C-terminal extension (Fig. S3). This was unexpected as, other than proteins containing the DbpA RNA binding domain, sequence conservation in the C-terminal extension of DEAD-box RNA helicases is generally minimal [17]. Therefore, it is likely that both the CrhR-like helicases and the DEAD-box protein from *G. violaceus* PCC 7421 share a common ancestral protein, with evolution of the CrhR-specific C-terminal motif occurring after the divergence of the order *Gloeobacterales *.

### CsdA-like proteins are characterized by a DbpA RNA binding domain in the C-terminal extension

**Fig. 5.**
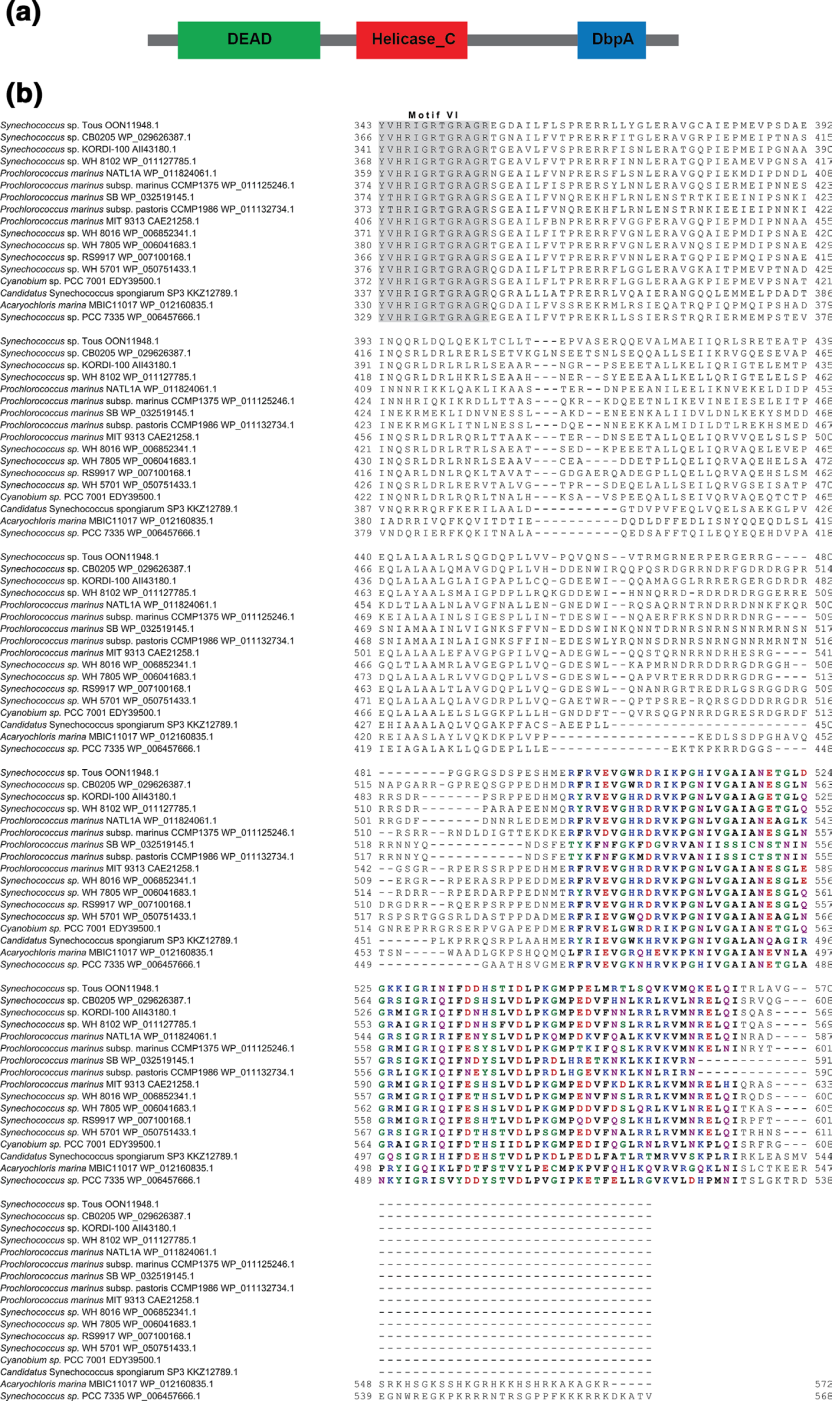
The C-terminal extension of the CsdA-like helicases contains a DbpA RNA binding domain. (**a**) A schematic showing the Pfam domains present in the CsdA-like helicases: the DEAD/DEAH box helicase domain (DEAD, PF00270), the helicase conserved C-terminal domain (helicase C, PF00271) and the DbpA RNA binding domain (DbpA, PF03880). (**b**) Alignments generated with muscle of the C-terminal extensions of selected CsdA-like helicases. The final conserved DEAD-box helicase motif, HRIGRXXR, is marked with a grey background on the alignment. The conserved DbpA RNA binding domain is indicated in bold, with amino acids coloured based on their chemical properties. Note that this domain is not present in members of the CrhR-like clade.

The DbpA RNA binding motif is an arginine-rich protein motif that facilitates recognition and binding of RNA, specifically hairpin 92 of the 23S rRNA [18–20]. As the *E. coli* DEAD-box RNA helicase CsdA, which has a DbpA RNA binding motif in its C-terminal extension (Fig. 4), clustered within the cyanobacterial CsdA-like clade, the C-terminal extension of helicases in this study were also examined for a DbpA RNA binding motif. The C-terminal extension of the CsdA-like DEAD-box RNA helicases extends 200–235 amino acids past the final motif of the helicase core region, the HRIGR motif VI (Fig. S1). It was found that in CsdA-like cyanobacterial helicases, the C-terminal extension contains a DbpA RNA binding motif, a domain that is not observed in the CrhR-like helicases (Fig. 5). Two other cyanobacterial helicases also contain a DbpA RNA binding motif in their C-terminus: proteins from the unclassified cyanobacteria bacterium strain 13_1_20 CM_4_61_6 (OLE96526.1) and *Hassallia byssoidea* VB512170 (KIF31317.1). These strains cluster near the CsdA-like proteins and *E. coli* DbpA, respectively, with low bootstrapping support, suggesting a more distant relationship within the same larger group of DbpA-containing DEAD-box proteins.

### RhlE-like helicases co-occur with another DEAD-box protein

**Fig. 6.**
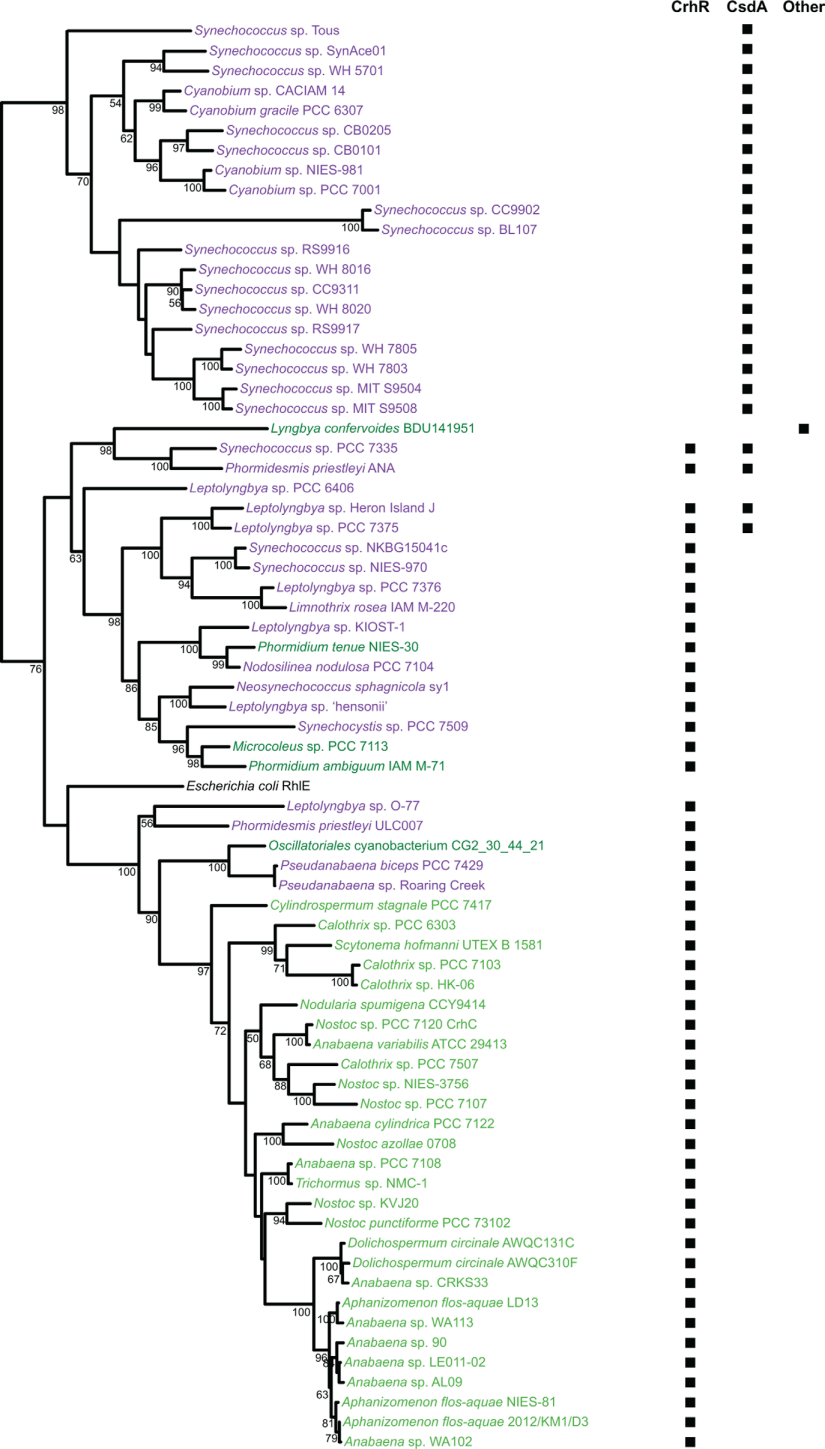
Co-occurrence of RhlE-like helicases with CsdA- and CrhR-like proteins. The RhlE-like maximum-likelihood helicase tree was extracted from Fig. S2. Annotations indicate whether the second DEAD-box protein in each strain is from the CrhR-like or CsdA-like groups. Essentially, RhlE is only found in conjunction with either CrhR or CsdA. Bootstrap values ≥50 % are shown at the nodes. Taxa colours indicate the cyanobacterial taxonomic order: purple, *Synechococcales *; light green, *Nostocales *; dark green, *Oscillatoriales *. The position of *E. coli* RhlE is provided to support the classification.

RhlE-like proteins essentially only occur in strains of cyanobacteria with at least one other DEAD-box protein (Fig. 6). *Leptolyngbya* sp. PCC 6406 is the only cyanobacterium in this study that encodes an RhlE-like helicase as its only identified DEAD-box protein (Table S1); however, as the genome sequence of this organism is a high-quality draft sequence, we predict that an additional DEAD-box RNA helicase, either CrhR- or CsdA-like, is encoded in the remaining gapped regions.

The RhlE-like proteins cluster into two major related groups that, apart from the four strains that encode all three types of cyanobacterial DEAD-box proteins, correlate with the co-occurrence of CsdA- or CrhR-like helicases (Fig. 6). This suggests that RhlE-like helicases were acquired at least twice in cyanobacterial lineages, once in the lineage with CsdA-like helicases and at least once in strains with CrhR-like proteins.

The RhlE-like proteins have a C-terminal extension of approximately 75–100 amino acids that lacks sequence conservation (Fig. 4). It should also be noted that all RhlE-like proteins from the order *Nostocales* had a substitution of phenylalanine for serine in the conserved motif III [62], resulting in a sequence change of SAT→FAT.

### Proteins from all three helicase clades are only encoded in four related strains of cyanobacteria

**Fig. 7.**
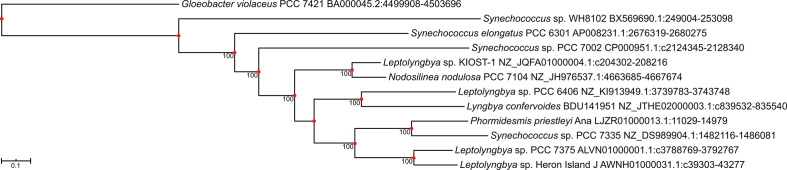
*rpoC2* maximum-likelihood tree showing that the four cyanobacterial strains that encode DEAD-box proteins from all three clades of cyanobacterial DEAD-box proteins are closely related. Nucleotide sequences for the RNA polymerase β′ subunit, *rpoC2*, were aligned by muscle and used to reconstruct a maximum-likelihood tree with *G. violaceus* PCC 7421 as an outgroup. Several *Leptolyngbya* species and closely related cyanobacteria, as well as several *Synechococcus* species, were included to confirm the clustering of the four strains that encode all three DEAD-box proteins. Bootstrap values ≥50 % are shown at the nodes. Branch lengths are proportional to the mean number of substitutions per nucleotide site as indicated by the scale bar. All nodes in the maximum-likelihood tree are duplicated in a neighbour-joining reconstruction, as indicated with a red circle.

The only cyanobacterial strains that encode a member of all three clades, a CrhR-, CsdA- and RhlE-like helicase, within the same genome are *Leptolyngbya* sp. PCC 7375*, Leptolyngbya* sp. Heron Island J, *Phormidesmis priestleyi* Ana and *Synechococcus* sp. PCC 7335 (Fig. S2). Although these four strains are annotated as members of three separate genera, 16S rDNA, *rpoC2* and/or concatenated gene phylogenies indicate they are more closely related than another closely related strain, *Leptolyngbya* sp. PCC 6406 [58, 63, 64]. As all four of these strains have not been included in the same phylogenetic analysis to date, an *rpoC2* gene tree was reconstructed (Fig. 7) to confirm that the strains were closely related. As expected, *Leptolyngbya* sp. PCC 7375, *Leptolyngbya* sp. Heron Island J, *Phormidesmis priestleyi* Ana and *Synechococcus* sp. PCC 7335 cluster together (Fig. 7). This clustering was also supported by 16S rDNA analysis in which all nodes were duplicated, excepting the node nearest *Phormidesmis priestleyi* Ana (Fig. S4), for which a 16S rDNA sequence is not available. Clustering of these four strains confirms that, despite the differences in cellular morphology and current taxonomy, the strains of cyanobacteria that encode members of all three groups of cyanobacterial DEAD-box proteins are closely related and require reclassification.

## Discussion

Cyanobacteria are unique among prokaryotes, as they are the only bacteria that perform oxygenic photosynthesis. In this study, it was shown that the complement of DEAD-box RNA helicases encoded by cyanobacteria is also unique, as proteins with homology to the helicases of *E. coli *, the CsdA-like and RhlE-like protein groups, are only encoded within the genomes of select cyanobacterial taxa. In contrast, the DEAD-box RNA helicase clade that can be found across cyanobacterial diversity, the CrhR-like proteins, is unique to cyanobacteria. The C-terminal extension domain of the DEAD-box RNA helicase is a defining characteristic of this unique clade.

A sequence motif of approximately 50 amino acids that is uniquely observed only in the CrhR-like clade of cyanobacterial DEAD-box RNA helicases is located within the C-terminal extension. This motif is not observed in other organisms, including higher plants and algae, but is widely distributed in cyanobacteria, suggesting that the CrhR-specific domain arose as a specialization of a DEAD-box protein in the ancestral cyanobacterial lineage. This implies that CrhR-like helicases perform a specific function that is unique to cyanobacteria. As CrhR-like helicases are often the sole DEAD-box protein in a cyanobacterial genome, it is likely that they function in multiple RNA processes. We propose that the unique function of CrhR-like helicases likely relates to oxygenic photosynthesis. This conjecture is supported by the co-precipitation of CrhR from *Synechocystis* with polysomes and RNA degradosome components [35], and the altered expression of ~10 % of the *Synechocystis* transcriptome, consisting of both mRNA and non-coding RNAs, in a *crhR* truncation mutant [65]. Physiologically, and consistent with the altered expression profiles, CrhR mutation results in significant disruption to photosynthesis, with cells exhibiting reduced pigment abundance, oxygen evolution rates and carbon dioxide fixation rates [36]. These physiological changes can also be associated with the regulation of *crhR* expression by a range of abiotic stresses [32, 34, 66, 67]. Taken together, these effects are consistent with CrhR RNA helicase localization to the thylakoid membrane [35], the site of light harvesting and ATP and NADPH formation in *Synechocystis *. Therefore, we propose that CrhR-like RNA helicases perform a unique function, coordinating expression of genes required to maintain oxygenic photosynthesis in response to abiotic stresses in cyanobacteria.

Support for CrhR functioning in maintenance of photosynthesis was also provided by the conserved gene synteny surrounding the CrhR-like proteins. The *rimO–crhR* operon of *Synechocystis* is conserved in 89.8 % of strains with CrhR-like helicases. Over 60 % of strains also have further conservation of synteny, with conserved genes relating to photosynthesis, energy transfer from the electron transport chain and membrane protein stability. In particular, the activities of many of these proteins could protect the photosynthetic electron transport chain from oxidative damage if an excess of reductant was to accumulate. For example, PsbU stabilizes the oxygen-evolving complex of photosystem II (PSII) and protects it from inactivation in response to stress, such as heat [68]. The vitamin K epoxide reductase (VKOR) and l-aspartate oxidase (*nadB*) act as electron sinks, as they both can use quinones from the electron transport chain as the source of energy for their biochemical activity [69, 70]. Expression of these proteins may be regulated under similar conditions as CrhR, which is induced in response to increasing reduction of the photosynthetic electron transport chain [34].

It is interesting that CrhR- and CsdA-like helicases are not observed in the same species, suggesting the requirement for divergent functionality. The CsdA-like helicases are characterized by a DbpA RNA binding domain in the C-terminus, similar to the canonical family member CsdA in *E. coli* [17]. The presence of the DbpA RNA binding domain, which assists DbpA in binding specifically to hairpin 92 of the 23S rRNA [18, 71], may indicate that the cyanobacterial CsdA-like helicases have a role in ribosome biogenesis, and more specifically maturation of the 23S rRNA, similar to DbpA [23, 72]. The domain may also provide more general RNA chaperone properties to the helicase, as some proteins with a DbpA RNA binding domain, including CsdA, do not bind to the 23S rRNA at hairpin 92 [73]. As these proteins have significant homology with CsdA, the cyanobacterial CsdA-like proteins may constitute a multifunctional helicase that can function in ribosome biogenesis, translation initiation and RNA degradation, as observed in *E. coli* [21, 23, 73–76]. The DbpA RNA binding domain is not encoded in CrhR-like helicases, suggesting CrhR is not involved in ribosome biogenesis.

Cyanobacterial RhlE-like helicases lack a conserved C-terminal domain. Interestingly, proteins from this group are essentially only encoded in genomes with at least one other DEAD-box helicase. This is consistent with the distribution of RhlE-like proteins generally in prokaryotes, with only a limited number of genomes encoding solely an RhlE-like DEAD-box helicase [17]. This also supports conservation of the proposed function of the RhlE helicase in *E. coli* as a helicase that functions cooperatively with other DEAD-box proteins during ribosome biogenesis [77].

Thus, distribution and sequence divergence of the CsdA and RhlE groups of DEAD-box RNA helicases in cyanobacteria suggest they were acquired independently several times. The CrhR-like proteins are widely distributed throughout cyanobacterial diversity and contain a sequence domain unique to the CrhR-like clade of DEAD-box RNA helicases, suggesting they perform a cyanobacterial specific function. Overall, the analysis suggests that the precursor of CrhR-like DEAD-box helicases arose early in cyanobacterial evolution. A common ancestor of the genera *Synechococcus* and *Prochlorococcus* likely lost the CrhR-like helicase. Early branching *Synechococcus* and *Prochlorococcus *, based on 16S rRNA sequence [78], contain CrhR-like helicases; however, CrhR-like proteins are absent from *Synechococcus elongatus* PCC 7942 and the strains with CsdA-like helicases. It is possible that the CsdA-like helicases were acquired by horizontal gene transfer in the ancestral lineage that lacked a CrhR-like helicase and were maintained as they conferred a selective advantage. Acquisition of RhlE-like helicases in cyanobacteria was likely also by horizontal gene transfer, possibly several events, as they are generally only found in proteobacteria [17].

This study raises questions as to why DEAD-box RNA helicases encoded in cyanobacterial genomes display distributions restricted on both the family member type and strain levels. This suggests certain lineages of cyanobacteria require the activity of specific DEAD-box RNA helicases. The specificity is potentially related to habitat niche for marine species, since CsdA-like helicases are principally encoded in *Synechococcus* and *Prochlorococcus* species, which are primarily marine; however, this does not apply to the CrhR-like helicases, which occur in cyanobacteria that occupy a diverse range of habitats. Cyanobacteria primarily encode helicases classified into three DEAD-box RNA helicase clades on the basis of conserved sequences in the C-terminal extension. Two of these clades, the CsdA-like and RhlE-like helicases, have significant similarity to the corresponding *E. coli* proteins. The third clade, the CrhR-like helicases, are unique to cyanobacteria and are characterized by a sequence motif in the C-terminal extension that is only found in CrhR-like helicases. Based on prior characterization of CrhR and the observed syntenic gene conservation, it is likely that CrhR-like proteins are multifunctional helicases, performing roles in RNA metabolism related to maintenance of photosynthesis.

## Supplementary Data

Supplementary material 1
