# Supplementary material for: Genera specific distribution of DEAD-box RNA helicases in cyanobacteria
Source: Microb Genom. 2021 Feb 4;7(3):mgen000517. doi: 10.1099/mgen.0.000517 (PMC8190605; doi:10.1099/mgen.0.000517)
Supplement: Supplementary material 1 [file mgen-7-0517-s001.pdf]

Supplementary Material

Genera specific distribution of DEAD-box RNA helicases in cyanobacteria

Denise S. Whitford, Brendan T. Whitman and George W. Owttrim

Department of Biological Sciences, University of Alberta, Edmonton, AB, Canada T6G 2E9

This file contains one supplementary table: **Table S1**, four supplementary figures: **Fig. S1**, **Fig. S2**, **Fig. S3** and **Fig. S4** and supplementary references.

Table S1. DEAD-box RNA helicase proteins encoded in cyanobacterial genomes. The list of sequence identifiers was obtained by searching the NCBI non-redundant sequences database using the helicase core domain (F9-R338) of the DEAD-box protein CrhR (*slr0083*) from *Synechocystis* sp. PCC 6803. The order, species, strain and accession number for each protein is indicated, as well as the corresponding clade (CrhR, CsdA or RhlE) within the DEAD-box protein family.

Order	Species	Strain	Accession Number	Group
Chroococcales				
	Aphanothece halophytica	PCC 7418	WP_015227798.1	CrhR
	Candidatus Atelocyanobacterium thalassa isolate	ALOHA	WP_012954034.1	CrhR
	Candidatus Atelocyanobacterium thalassa isolate	SIO64986	KFF41262.1	CrhR
	Chroogloeocystis siderophila	5.2 s.c.1	WP_073551513.1	CrhR
	Crocosphaera watsonii	WH 8501	WP_007303466.1	CrhR
	Cyanobacterium aponinum	PCC 10605	WP_015219980.1	CrhR
	Cyanobacterium sp.	IPPAS B-1200	WP_069789285.1	CrhR
	Cyanobacterium stanieri	PCC 7202	AFZ47545.1	CrhR
	Geminocystis herdmanii	PCC 6308	WP_017294180.1	CrhR
	Geminocystis sp.	NIES-3708	WP_066348995.1	CrhR
	Geminocystis sp.	NIES-3709	WP_066117391.1	CrhR
	Gloeocapsa sp.	PCC 7428	WP_015190184.1	CrhR
	Gloeocapsa sp.	PCC 73106	WP_006530822.1	CrhR
	Microcystis aeruginosa	KW	WP_079208639.1	CrhR
	Microcystis aeruginosa	NIES-44	WP_045361726.1	CrhR
	Microcystis aeruginosa	NIES-88	WP_061432786.1	CrhR
	Microcystis aeruginosa	NIES-98	WP_069474107.1	CrhR
	Microcystis aeruginosa	NIES-843	WP_012265913.1	CrhR
	Microcystis aeruginosa	NIES-2481	WP_066030023.1	CrhR
	Microcystis aeruginosa	NIES-2549	WP_046662813.1	CrhR
	Microcystis aeruginosa	PCC 7005	WP_024970412.1	CrhR
	Microcystis aeruginosa	PCC 7806	WP_002741230.1	CrhR
	Microcystis aeruginosa	PCC 9432	WP_002733094.1	CrhR
	Microcystis aeruginosa	PCC 9443	WP_002769059.1	CrhR
	Microcystis aeruginosa	PCC 9701	WP_004268339.1	CrhR
	Microcystis aeruginosa	PCC 9717	WP_002763242.1	CrhR
	Microcystis aeruginosa	PCC 9806	WP_002781592.1	CrhR
	Microcystis aeruginosa	SPC777	WP_016516037.1	CrhR
	Microcystis panniformis	FACHB-1757	WP_052277776.1	CrhR
	Microcystis sp.	T1-4	WP_008198520.1	CrhR
	Rubidibacter lacunae	KORDI 51-2	WP_022606644.1	CrhR

Order	Species	Strain	Accession Number	Group
Chroococcidiopsidales				
	Chroococcidiopsis thermalis	PCC 7203	WP_015155494.1	CrhR
Gloeobacterales				
	Gloeobacter kilaueensis	JS1	WP_023172363.1	No group
	Gloeobacter violaceus	PCC 7421 (a)	WP_011142503.1	No group
	Gloeobacter violaceus	PCC 7421 (b)	WP_011142321.1	No group
Nostocales				
	Anabaena cylindrica	PCC 7122	WP_015216841.1	CrhR
	Anabaena cylindrica	PCC 7122	WP_015217396.1	RhlE
	Anabaena sp.	90	WP_015081159.1	CrhR
	Anabaena sp.	90	WP_015079554.1	RhlE
	Anabaena sp.	AL09	OBQ05623.1	CrhR
	Anabaena sp.	AL09	OBQ03207.1	RhlE
	Anabaena sp.	ATCC 33047	WP_066377041.1	CrhR
	Anabaena sp.	CRKS33	OBQ40729.1	CrhR
	Anabaena sp.	CRKS33	OBQ32742.1	RhlE
	Anabaena sp.	LE011-02	OBQ04690.1	CrhR
	Anabaena sp.	LE011-02	OBQ05997.1	RhlE
	Anabaena sp.	PCC 7108	WP_016953324.1	CrhR
	Anabaena sp.	PCC 7108	WP_016951584.1	RhlE
	Anabaena sp.	WA102	WP_053540454.1	CrhR
	Anabaena sp.	WA102	WP_053539369.1	RhlE
	Anabaena sp.	WA113	OBQ22342.1	CrhR
	Anabaena sp.	WA113	OBQ18746.1	RhlE
	Anabaena variabilis	ATCC 29413	ABA20266.1	CrhR
	Anabaena variabilis	ATCC 29413	ABA21574.1	RhlE
	Aphanizomenon flos-aquae	2012/KM1/D3	WP_039204634.1	CrhR
	Aphanizomenon flos-aquae	2012/KM1/D3	WP_039200299.1	RhlE
	Aphanizomenon flos-aquae	LD13	OBQ18590.1	CrhR
	Aphanizomenon flos-aquae	LD13	OBQ22990.1	RhlE
	Aphanizomenon flos-aquae	NIES-81	WP_027404570.1	CrhR
	Aphanizomenon flos-aquae	NIES-81	WP_027401622.1	RhlE
	Calothrix sp.	336/3	WP_035153586.1	CrhR
	Calothrix sp.	HK-06	WP_073618868.1	CrhR
	Calothrix sp.	HK-06	WP_073622274.1	RhlE
	Calothrix sp.	PCC 6303	WP_015200162.1	CrhR
	Calothrix sp.	PCC 6303	WP_015195959.1	RhlE
	Calothrix sp.	PCC 7103	WP_019489262.1	CrhR
	Calothrix sp.	PCC 7103	WP_035173265.1	RhlE
	Calothrix sp.	PCC 7507	WP_015131393.1	CrhR
	Calothrix sp.	PCC 7507	WP_015132072.1	RhlE
	Chlorogloeopsis fritschii	PCC 6912	WP_016873588.1	CrhR
	Chlorogloeopsis sp.	PCC 7702	WP_017322836.1	CrhR

Order	Species	Strain	Accession Number	Group
Nostocales				
	Chrysosporum ovalisporum	UAM-MAO	CEJ43554.1	CrhR
	Cylindrospermopsis raciborskii	CS-505	WP_006276327.1	CrhR
	Cylindrospermopsis raciborskii	MVCC14	WP_071250936.1	CrhR
	Cylindrospermopsis sp.	CR12	WP_057176886.1	CrhR
	Cylindrospermum stagnale	PCC 7417	WP_015206929.1	CrhR
	Cylindrospermum stagnale	PCC 7417	WP_015209745.1	RhlE
	Dolichospermum circinale	AWQC131C	WP_028090653.1	CrhR
	Dolichospermum circinale	AWQC131C	WP_028091112.1	RhlE
	Dolichospermum circinale	AWQC310F	WP_028084471.1	CrhR
	Dolichospermum circinale	AWQC310F	WP_028084089.1	RhlE
	Fischerella major	NIES-592	WP_073555827.1	CrhR
	Fischerella muscicola	PCC 73103	WP_016860456.1	CrhR
	Fischerella muscicola	PCC 7414	WP_026085820.1	CrhR
	Fischerella sp.	JSC-11	WP_009453863.1	CrhR
	Fischerella sp.	NIES-3754	WP_062246540.1	CrhR
	Fischerella sp.	PCC 9339	WP_017311292.1	CrhR
	Fischerella sp.	PCC 9605	WP_026731393.1	CrhR
	Fortiea contorta	PCC 7126	WP_017654089.1	CrhR
	Hapalosiphon sp.	MRB220	WP_026721223.1	CrhR
	Hassallia byssoidea	VB512170	KIF28888.1	CrhR
	Hassallia byssoidea	VB512170 (a)	KIF30245.1	No group
	Hassallia byssoidea	VB512170 (b)	KIF31317.1	No group
	Mastigocladopsis repens	PCC 10914	WP_017315850.1	CrhR
	Mastigocoleus testarum	BC008	WP_027842873.1	CrhR
	Nodularia spumigena	CCY9414	WP_006195458.1	CrhR
	Nodularia spumigena	CCY9414	WP_006195497.1	RhlE
	Nodularia spumigena	CENA596	WP_063873820.1	CrhR
	Nostoc azollae	0708	WP_013190002.1	CrhR
	Nostoc azollae	0708	ADI63752.1	RhlE
	Nostoc calcicola	FACHB-389	WP_073642635.1	CrhR
	Nostoc piscinale	CENA21	WP_062295266.1	CrhR
	Nostoc punctiforme	PCC 73102	WP_012410842.1	CrhR
	Nostoc punctiforme	PCC 73102	WP_012409963.1	RhlE
	Nostoc sp.	KVJ20	WP_069069063.1	CrhR
	Nostoc sp.	KVJ20	WP_069072171.1	RhlE
	Nostoc sp.	MBR 210	OCQ99908.1	CrhR
	Nostoc sp.	NIES-3756	WP_067773294.1	CrhR
	Nostoc sp.	NIES-3756	WP_067768693.1	RhlE
	Nostoc sp.	PCC 7107	WP_015111687.1	CrhR
	Nostoc sp.	PCC 7107	AFY45650.1	RhlE
	Nostoc sp.	PCC 7120 (CrhB)	WP_010995395.1	CrhR
	Nostoc sp.	PCC 7120 (CrhC)	WP_010998849.1	RhlE
	Nostoc sp.	PCC 7524	WP_015138780.1	CrhR
	Raphidiopsis brookii	D9	WP_009343384.1	CrhR

Order	Species	Strain	Accession Number	Group
Nostocales				
	Richelia intracellularis	HH01	WP_008231885.1	CrhR
	Richelia intracellularis	RC01	CDN14942.1	CrhR
	Rivularia sp.	PCC 7116	WP_015121796.1	CrhR
	Scytonema hofmanni	UTEX B 1581	WP_029631515.1	RhlE
	Scytonema hofmanni	UTEX B 1581	WP_029633835.1	CrhR
	Scytonema hofmannii	PCC 7110	WP_017746800.1	CrhR
	Scytonema sp.	HK-05	WP_073628368.1	CrhR
	Scytonema tolypothrichoides	VB-61278	WP_048871842.1	CrhR
	Tolypothrix bouteillei	VB521301	WP_038078122.1	CrhR
	Tolypothrix campylonemoides	VB511288	WP_071838559.1	No group
	Tolypothrix campylonemoides	VB511288	WP_041035416.1	CrhR
	Tolypothrix sp.	PCC 7601	WP_045867700.1	CrhR
	Trichormus sp.	NMC-1	WP_071190055.1	CrhR
	Trichormus sp.	NMC-1	WP_071190771.1	RhlE
Oscillatoriales				
	Arthrospira maxima	CS-328	WP_006670629.1	CrhR
	Arthrospira platensis	C1	WP_035760177.1	CrhR
	Arthrospira platensis	NIES-39	WP_043468916.1	CrhR
	Arthrospira platensis	Paraca	WP_006617243.1	CrhR
	Coleofasciculus chthonoplastes	PCC 7420	WP_006102098.1	CrhR
	Crinalium epipsammum	PCC 9333	WP_015205239.1	CrhR
	Cyanothece sp.	ATCC 51142	WP_009543508.1	CrhR
	Cyanothece sp.	CCY0110	WP_008276945.1	CrhR
	Cyanothece sp.	PCC 7425	WP_012629831.1	CrhR
	Cyanothece sp.	PCC 7822	WP_013323350.1	CrhR
	Cyanothece sp.	PCC 8801	WP_015785253.1	CrhR
	Desertifilum sp.	IPPAS B-1220	WP_069967535.1	CrhR
	filamentous cyanobacterium	ESFC-1	WP_018396245.1	CrhR
	Geitlerinema sp.	PCC 7105	WP_017661231.1	No group
	Geitlerinema sp.	PCC 7407	WP_015173091.1	CrhR
	Geitlerinema sp.	PCC 9228	WP_071517408.1	CrhR
	Limnoraphis robusta	CS-951	WP_046278382.1	CrhR
	Lyngbya aestuarii	BL J	WP_023065801.1	CrhR
	Lyngbya confervoides	BDU141951	WP_044150923.1	RhlE
	Lyngbya confervoides	BDU141951	WP_063776105.1	No group
	Lyngbya sp.	PCC 8106	WP_039896992.1	CrhR
	Microcoleus sp.	PCC 7113	WP_015182542.1	RhlE
	Microcoleus sp.	PCC 7113	WP_015184396.1	CrhR
	Microcoleus vaginatus	FGP-2	WP_006633595.1	CrhR
	Moorea bouillonii	PNG5-198	WP_075900005.1	CrhR
	Moorea producens	3L	WP_008177600.1	CrhR
	Moorea producens	JHB	WP_071104591.1	CrhR
	Moorea producens	PAL-8-15-08-1	WP_070393209.1	CrhR

Order	Species	Strain	Accession Number	Group
Oscillatoriales				
	Oscillatoria acuminata	PCC 6304	WP_015146953.1	CrhR
	Oscillatoria nigro-viridis	PCC 7112	WP_015177386.1	CrhR
	Oscillatoria sp.	PCC 10802	WP_017715337.1	CrhR
	Oscillatoria sp.	PCC 6506	WP_007354567.1	CrhR
	Oscillatoriales cyanobacterium	CG2_30_40_61	OIP73250.1	CrhR
	Oscillatoriales cyanobacterium	CG2_30_44_21	OIP71395.1	CrhR
	Oscillatoriales cyanobacterium	CG2_30_44_21	OIP71446.1	RhlE
	Oscillatoriales cyanobacterium	JSC-12	WP_009557052.1	CrhR
	Oscillatoriales cyanobacterium	MTP1	WP_058883613.1	CrhR
	Oscillatoriales cyanobacterium	USR001	OCR00684.1	CrhR
	Phormidium ambiguum	IAM M-71	WP_073595642.1	RhlE
	Phormidium ambiguum	IAM M-71	WP_073595739.1	CrhR
	Phormidium sp.	OSCR	KPQ34456.1	No group
	Phormidium tenue	NIES-30	WP_073610295.1	RhlE
	Phormidium tenue	NIES-30	WP_073607089.1	CrhR
	Planktothricoides sp.	SR001	WP_054466936.1	CrhR
	Planktothrix agardhii	NIVA-CYA 126/8	WP_042151026.1	CrhR
	Planktothrix agardhii	PCC 7805	CUM60384.1	CrhR
	Planktothrix paucivesiculata	PCC 9631	CUR15004.1	CrhR
	Planktothrix rubescens	7821	WP_026787128.1	CrhR
	Planktothrix serta	PCC 8927	CUR16751.1	CrhR
	Planktothrix sp.	PCC 11201	WP_079680322.1	CrhR
	Planktothrix tepida	PCC 9214	WP_072718935.1	CrhR
	Roseofilum reptotaenium	AO1-A	OJJ22692.1	CrhR
	Trichodesmium erythraeum	IMS101	ABG52788.1	CrhR
Pleurocapsales				
	Hydrococcus rivularis	NIES-593	WP_073598826.1	CrhR
	Myxosarcina sp.	GI1	WP_036485380.1	CrhR
	Pleurocapsa sp.	PCC 7319	WP_019508420.1	CrhR
	Pleurocapsa sp.	PCC 7327	WP_015145756.1	CrhR
	Stanieria cyanosphaera	PCC 7437	WP_015194510.1	CrhR
	Stanieria sp.	NIES-3757	BAU65148.1	CrhR
	Xenococcus sp.	PCC 7305	WP_006508547.1	CrhR
Spirulinales				
	Spirulina major	PCC 6313	WP_072621974.1	CrhR
	Spirulina subsalsa	PCC 9445	WP_017305715.1	CrhR
Synechococcales				
	Acaryochloris marina	MBIC11017	WP_012162240.1	CrhR
	Acaryochloris marina	MBIC11017	WP_012160835.1	CsdA
	Acaryochloris sp.	CCMEE 5410	WP_010476696.1	CrhR
	Acaryochloris sp.	CCMEE 5410	WP_010469002.1	CsdA

Order	Species	Strain	Accession Number	Group
Synechococcales				
	Aphanocapsa montana	BDHKU210001 (a)	KIF26984.1	No group
	Aphanocapsa montana	BDHKU210001 (b)	KIF27867.1	No group
	Candidatus Synechococcus spongiarum		WP_074457638.1	CsdA
	Candidatus Synechococcus spongiarum	142	KKZ10452.1	CsdA
	Candidatus Synechococcus spongiarum	LMB bulk15M	OOV28380.1	CsdA
	Candidatus Synechococcus spongiarum	SH4	WP_025782217.1	CsdA
	Candidatus Synechococcus spongiarum	SP3	KKZ12789.1	CsdA
	Chamaesiphon minutus	PCC 6605	AFY93922.1	CrhR
	Cyanobium gracile	PCC 6307	AFY27888.1	RhlE
	Cyanobium gracile	PCC 6307	WP_015108547.1	CsdA
	Cyanobium sp.	CACIAM 14	KEF42712.1	RhlE
	Cyanobium sp.	CACIAM 14	KEF42202.1	CsdA
	Cyanobium sp.	NIES-981	SBO41936.1	RhlE
	Cyanobium sp.	NIES-981	SBO42872.1	CsdA
	Cyanobium sp.	PCC 7001	WP_006910544.1	RhlE
	Cyanobium sp.	PCC 7001	EDY39500.1	CsdA
	Dactylococcopsis salina	PCC 8305	WP_015230714.1	CrhR
	Leptolyngbya boryana	IAM M-101	WP_036045359.1	CrhR
	Leptolyngbya sp.	'hensonii'	WP_075596736.1	CrhR
	Leptolyngbya sp.	'hensonii'	WP_075601042.1	RhlE
	Leptolyngbya sp.	Heron Island J	WP_023075084.1	RhlE
	Leptolyngbya sp.	Heron Island J	WP_023073685.1	CrhR
	Leptolyngbya sp.	Heron Island J	WP_036052321.1	CsdA
	Leptolyngbya sp.	JSC-1	WP_036003541.1	CrhR
	Leptolyngbya sp.	KIOST-1	WP_035986653.1	RhlE
	Leptolyngbya sp.	KIOST-1	WP_035986580.1	CrhR
	Leptolyngbya sp.	NIES-2104	WP_058994620.1	CrhR
	Leptolyngbya sp.	NIES-3755	WP_068384456.1	CrhR
	Leptolyngbya sp.	O-77	BAU42382.1	CrhR
	Leptolyngbya sp.	O-77	BAU44142.1	RhlE
	Leptolyngbya sp.	PCC 6406	WP_008312463.1	RhlE
	Leptolyngbya sp.	PCC 7375	WP_006514332.1	RhlE
	Leptolyngbya sp.	PCC 7375	WP_006515861.1	CrhR
	Leptolyngbya sp.	PCC 7375	WP_006517045.1	CsdA
	Leptolyngbya sp.	PCC 7376	WP_015132458.1	RhlE
	Leptolyngbya sp.	PCC 7376	WP_015135334.1	CrhR
	Leptolyngbya valderiana	BDU 20041 (a)	OAB57648.1	No group
	Leptolyngbya valderiana	BDU 20041 (b)	WP_063718621.1	No group
	Limnothrix rosea	IAM M-220	WP_075890464.1	RhlE
	Limnothrix rosea	IAM M-220	WP_075892628.1	CrhR
	Limnothrix sp.	CACIAM 69d	OKY70165.1	CrhR
	Limnothrix sp.	P13C2	OCQ91779.1	CrhR
	Neosynechococcus sphagnicola	sy1	WP_052128427.1	RhlE
	Neosynechococcus sphagnicola	sy1	WP_052128892.1	CrhR

Order	Species	Strain	Accession Number	Group
Synechococcales				
	Nodosilinea nodulosa	PCC 7104	WP_017300039.1	RhlE
	Nodosilinea nodulosa	PCC 7104	WP_017300013.1	CrhR
	Phormidesmis priestleyi	Ana	KPQ31693.1	CsdA
	Phormidesmis priestleyi	Ana	KPQ37420.1	RhlE
	Phormidesmis priestleyi	Ana	KPQ34081.1	CrhR
	Phormidesmis priestleyi	BC1401	WP_068814569.1	CrhR
	Phormidesmis priestleyi	ULC007	WP_073069542.1	CrhR
	Phormidesmis priestleyi	ULC007	WP_073074629.1	RhlE
	Prochlorococcus marinus	AS9601	WP_011818638.1	CsdA
	Prochlorococcus marinus	GP2	WP_032524576.1	CsdA
	Prochlorococcus marinus	MIT 1312	KZR62459.1	CsdA
	Prochlorococcus marinus	MIT 1313	KZR69712.1	CsdA
	Prochlorococcus marinus	MIT 1320	KZR74671.1	CsdA
	Prochlorococcus marinus	MIT 1323	KZR76670.1	CsdA
	Prochlorococcus marinus	MIT 1342	KZR84025.1	CsdA
	Prochlorococcus marinus	MIT 9107	WP_032514218.1	CsdA
	Prochlorococcus marinus	MIT 9201	WP_032522590.1	CsdA
	Prochlorococcus marinus	MIT 9202	WP_002807683.1	CsdA
	Prochlorococcus marinus	MIT 9211	WP_012195637.1	CsdA
	Prochlorococcus marinus	MIT 9215	WP_012007924.1	CsdA
	Prochlorococcus marinus	MIT 9301	WP_011863159.1	CsdA
	Prochlorococcus marinus	MIT 9302	WP_032527473.1	CsdA
	Prochlorococcus marinus	MIT 9303	ABM77716.1	CsdA
	Prochlorococcus marinus	MIT 9312	WP_011376662.1	CsdA
	Prochlorococcus marinus	MIT 9313	CAE21258.1	CsdA
	Prochlorococcus marinus	MIT 9314	WP_032515082.1	CsdA
	Prochlorococcus marinus	MIT 9321	WP_032516671.1	CsdA
	Prochlorococcus marinus	MIT 9322	WP_032518542.1	CsdA
	Prochlorococcus marinus	MIT 9515	WP_011820499.1	CsdA
	Prochlorococcus marinus	NATL1A	WP_011824061.1	CsdA
	Prochlorococcus marinus	NATL2A	WP_011294736.1	CsdA
	Prochlorococcus marinus	PAC1	WP_036906556.1	CsdA
	Prochlorococcus marinus	SB	WP_032519145.1	CsdA
	Prochlorococcus marinus	SCGC AAA795-F05	WP_075508085.1	CsdA
	Prochlorococcus marinus	SCGC AAA795-I15	WP_075448480.1	CsdA
	Prochlorococcus marinus	SCGC AAA795-J16	WP_075487375.1	CsdA
	Prochlorococcus marinus	SCGC AAA795-M23	WP_075438860.1	CsdA
	Prochlorococcus marinus subsp. marinus	CCMP1375	WP_011125246.1	CsdA
	Prochlorococcus marinus subsp. pastoris	CCMP1986	WP_011132734.1	CsdA
	Prochlorococcus sp.	HOT208_60m_805A16	WP_079293733.1	CsdA
	Prochlorococcus sp.	HOT208_60m_808G21	WP_079292895.1	CsdA
	Prochlorococcus sp.	HOT208_60m_808M21	WP_079339458.1	CsdA
	Prochlorococcus sp.	HOT208_60m_810B23	WP_079300013.1	CsdA
	Prochlorococcus sp.	HOT208_60m_813B04	WP_079331862.1	CsdA

Order	Species	Strain	Accession Number	Group
Synechococcales				
	Prochlorococcus sp.	HOT208_60m_813E23	WP_079334503.1	CsdA
	Prochlorococcus sp.	HOT208_60m_813G15	WP_079322771.1	CsdA
	Prochlorococcus sp.	HOT208_60m_813I02	WP_079330881.1	CsdA
	Prochlorococcus sp.	HOT208_60m_813O14	WP_079317072.1	CsdA
	Prochlorococcus sp.	HOT212_60m_824E10	WP_079342754.1	CsdA
	Prochlorococcus sp.	MIT 0601	KGG12263.1	CsdA
	Prochlorococcus sp.	MIT 0602	WP_036917918.1	CsdA
	Prochlorococcus sp.	MIT 0604	WP_042850581.1	CsdA
	Prochlorococcus sp.	MIT 0701	KGG25182.1	CsdA
	Prochlorococcus sp.	MIT 0801	WP_038653462.1	CsdA
	Prochlorococcus sp.	MIT 1303	KZR65207.1	CsdA
	Prochlorococcus sp.	MIT 1306	KZR62829.1	CsdA
	Prochlorococcus sp.	RS50	WP_077142451.1	CsdA
	Prochlorococcus sp.	SS52	WP_036974731.1	CsdA
	Pseudanabaena biceps	PCC 7429	WP_009629066.1	CrhR
	Pseudanabaena biceps	PCC 7429	WP_009629206.1	RhlE
	Pseudanabaena sp.	PCC 6802	WP_019503121.1	CrhR
	Pseudanabaena sp.	PCC 7367	WP_015166541.1	CrhR
	Pseudanabaena sp.	Roaring Creek	WP_055074499.1	CrhR
	Pseudanabaena sp.	Roaring Creek	WP_055074844.1	RhlE
	Synechococcus sp.	BL107	WP_050749884.1	RhlE
	Synechococcus sp.	BL107	WP_009789521.1	CsdA
	Synechococcus sp.	CB0101	WP_050778783.1	RhlE
	Synechococcus sp.	CB0101	WP_043716891.1	CsdA
	Synechococcus sp.	CB0205	WP_029626348.1	RhlE
	Synechococcus sp.	CB0205	WP_029626387.1	CsdA
	Synechococcus sp.	CC9311	WP_011619228.1	RhlE
	Synechococcus sp.	CC9311	WP_011618985.1	CsdA
	Synechococcus sp.	CC9605	ABB35397.1	CsdA
	Synechococcus sp.	CC9616	WP_028951803.1	CsdA
	Synechococcus sp.	CC9902	WP_011360193.1	CsdA
	Synechococcus sp.	CC9902	WP_071818395.1	RhlE
	Synechococcus sp.	KORDI-49	WP_043691566.1	CsdA
	Synechococcus sp.	KORDI-52	WP_038557033.1	CsdA
	Synechococcus sp.	KORDI-100	AII43180.1	CsdA
	Synechococcus sp.	MIT S9504	KZR88066.1	RhlE
	Synechococcus sp.	MIT S9504	WP_067325214.1	CsdA
	Synechococcus sp.	MIT S9508	WP_067097210.1	RhlE
	Synechococcus sp.	MIT S9508	WP_067096793.1	CsdA
	Synechococcus sp.	MIT S9509	WP_066905108.1	CsdA
	Synechococcus sp.	NIES-970	BAW96345.1	RhlE
	Synechococcus sp.	NIES-970	BAW95895.1	CrhR
	Synechococcus sp.	NKBG15041c	WP_024544561.1	RhlE
	Synechococcus sp.	NKBG15041c	WP_024544991.1	CrhR

Order	Species	Strain	Accession Number	Group
Synechococcales				
	Synechococcus sp.	PCC 7002	WP_012306248.1	CrhR
	Synechococcus sp.	PCC 7003	WP_065713301.1	CrhR
	Synechococcus sp.	PCC 7335	WP_038016552.1	RhlE
	Synechococcus sp.	PCC 7335	WP_006457592.1	CrhR
	Synechococcus sp.	PCC 7335	WP_006457666.1	CsdA
	Synechococcus sp.	PCC 7336	WP_017325424.1	CsdA
	Synechococcus sp.	PCC 7502	WP_015169589.1	CrhR
	Synechococcus sp.	RCC307	WP_011935977.1	CsdA
	Synechococcus sp.	RS9916	WP_007097992.1	RhlE
	Synechococcus sp.	RS9916	WP_038023305.1	CsdA
	Synechococcus sp.	RS9917	WP_050752552.1	RhlE
	Synechococcus sp.	RS9917	WP_007100168.1	CsdA
	Synechococcus sp.	SynAce01	WP_071800114.1	RhlE
	Synechococcus sp.	SynAce01	WP_071799904.1	CsdA
	Synechococcus sp.	Tous	OON11948.1	CsdA
	Synechococcus sp.	Tous	OON11565.1	RhlE
	Synechococcus sp.	WH 5701	WP_050751433.1	CsdA
	Synechococcus sp.	WH 5701	WP_006170577.1	RhlE
	Synechococcus sp.	WH 7803	WP_050815880.1	RhlE
	Synechococcus sp.	WH 7803	WP_011933414.1	CsdA
	Synechococcus sp.	WH 7805	EAR18874.1	RhlE
	Synechococcus sp.	WH 7805	WP_006041683.1	CsdA
	Synechococcus sp.	WH 8016	WP_038013123.1	RhlE
	Synechococcus sp.	WH 8016	WP_006852341.1	CsdA
	Synechococcus sp.	WH 8020	WP_048348119.1	RhlE
	Synechococcus sp.	WH 8020	WP_048346274.1	CsdA
	Synechococcus sp.	WH 8102	WP_011127785.1	CsdA
	Synechococcus sp.	WH 8103	WP_049691925.1	CsdA
	Synechococcus sp.	WH 8109	AHF63353.1	CsdA
	Synechocystis sp.	PCC 6714	AIE75077.1	CrhR
	Synechocystis sp.	PCC 6803 (CrhR)	BAA10556.1	CrhR
	Synechocystis sp.	PCC 7509	WP_009633465.1	RhlE
	Synechocystis sp.	PCC 7509	WP_009631343.1	CrhR
Unclassified				
	Cyanobacteria bacterium	13_1_20CM_4_61_6	OLE96526.1	No group
	cyanobacterium endosymbiont of *Epithemia turgida*		WP_044107193.1	CrhR
Enterobacterales				
	Escherichia coli	K-12	WP_001295553.1	CsdA
	Escherichia coli	K-12	WP_000123737.1	DbpA
	Escherichia coli	K-12	WP_000047499.1	RhlB
	Escherichia coli	K-12	WP_000007101.1	RhlE
	Escherichia coli	K-12	WP_000219193.1	SrmB

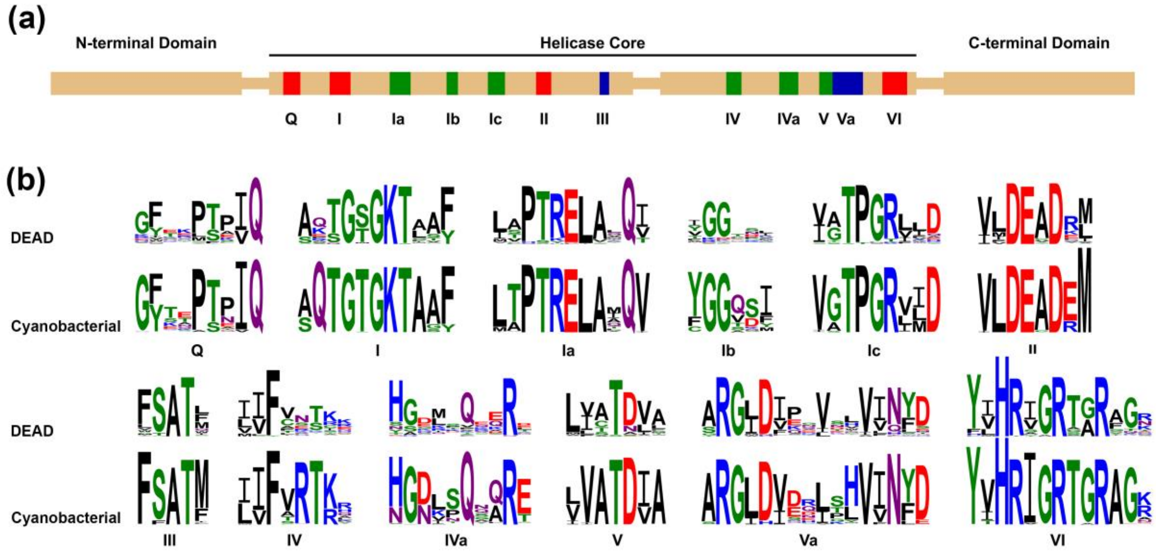

Fig. S1. Characteristic sequence motifs of DEAD-box RNA helicases. (a) Conserved domains characterizing the DEAD-box RNA helicase subfamily within SF2. The helicase core region consists of twelve conserved sequence motifs distributed within two recombinase A (RecA)-like domains. Accessory domains at the N- or C-terminus of the helicase core confer additional functionality or sequence specificity. Colours indicate the primary function of each motif: red for ATP binding and hydrolysis, green for RNA binding, and blue for coordination between ATP and RNA binding. (b) Sequence logos show conservation of the twelve motifs that characterize the DEAD-box protein family [1] with the 362 cyanobacterial DEAD-box helicases analyzed in this study. Sequence logos for the DEAD-box protein family were generated from alignments of the DEAD-box helicase complements of *Escherichia coli* (5) [2], *Bacillus subtilis* (4) [3], *Saccharomyces cerevisiae* (25) [4] and *Homo sapiens* (42) [5].

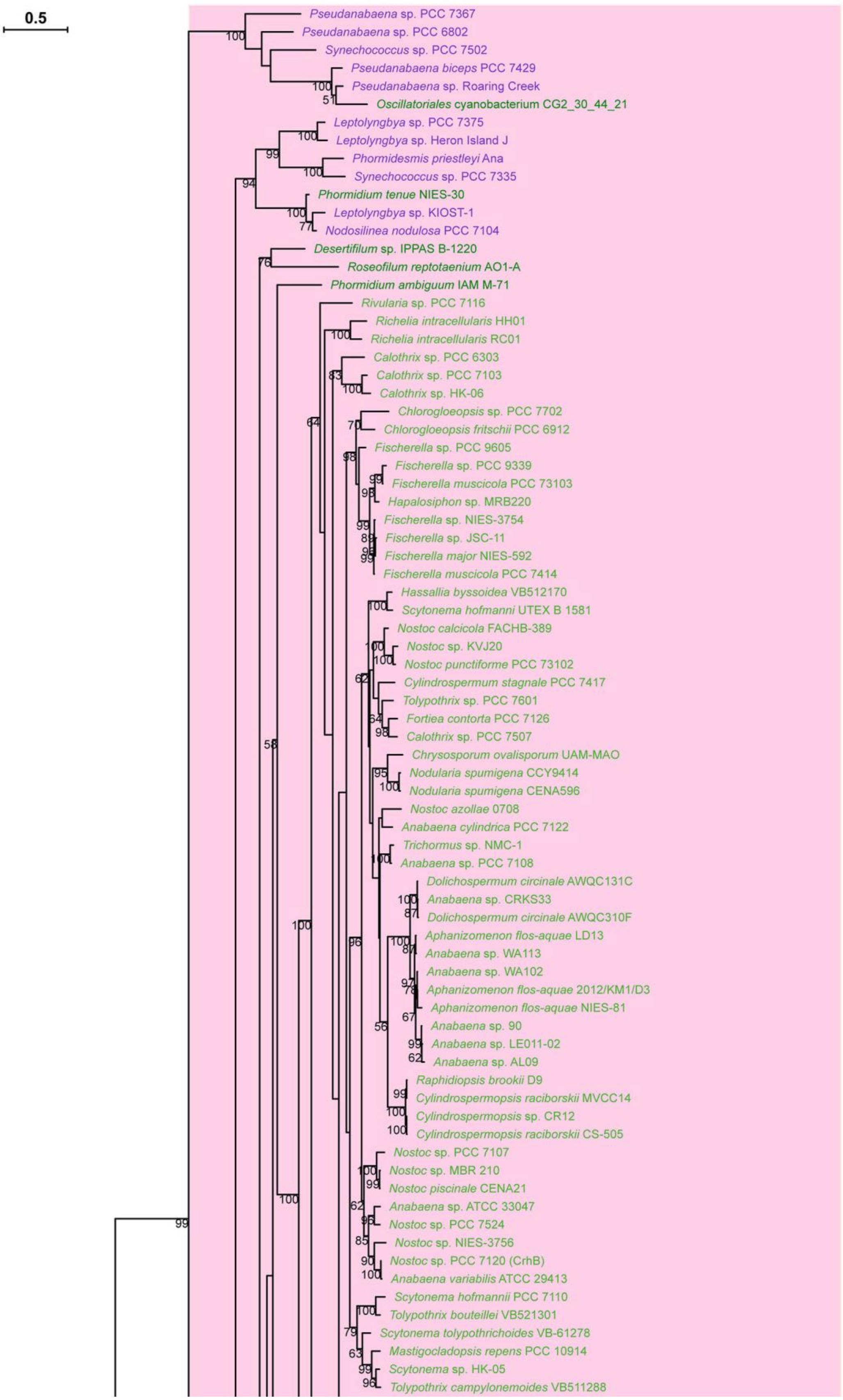
0.5
Pseudanabaena sp. PCC 7367
Pseudanabaena sp. PCC 6802
Synechococcus sp. PCC 7502
Pseudanabaena biceps PCC 7429
Pseudanabaena sp. Roaring Creek
Oscillatoriales cyanobacterium CG2_30_44_21
Leptolyngbya sp. PCC 7375
Leptolyngbya sp. Heron Island J
Phormidesmis priestleyi Ana
Synechococcus sp. PCC 7335
Phormidium tenue NIES-30
Leptolyngbya sp. KIOST-1
Nodosilinea nodulosa PCC 7104
Desertifilum sp. IPPAS B-1220
Roseofilum reptotaenium AO1-A
Phormidium ambiguum IAM M-71
Rivularia sp. PCC 7116
Richelia intracellularis HH01
Richelia intracellularis RC01
Calothrix sp. PCC 6303
Calothrix sp. PCC 7103
Calothrix sp. HK-06
Chlorogloeopsis sp. PCC 7702
Chlorogloeopsis fritschii PCC 6912
Fischerella sp. PCC 9605
Fischerella sp. PCC 9339
Fischerella muscicola PCC 73103
Hapalosiphon sp. MRB220
Fischerella sp. NIES-3754
Fischerella sp. JSC-11
Fischerella major NIES-592
Fischerella muscicola PCC 7414
Hassallia byssoidea VB512170
Scytonema hofmanni UTEX B 1581
Nostoc calcicola FACHB-389
Nostoc sp. KVJ20
Nostoc punctiforme PCC 73102
Cylindrospermum stagnale PCC 7417
Tolypothrix sp. PCC 7601
Fortiea contorta PCC 7126
Calothrix sp. PCC 7507
Chrysosporum ovalisporum UAM-MAO
Nodularia spumigena CCY9414
Nodularia spumigena CENA596
Nostoc azollae 0708
Anabaena cylindrica PCC 7122
Trichormus sp. NMC-1
Anabaena sp. PCC 7108
Dolichospermum circinale AWQC131C
Anabaena sp. CRKS33
Dolichospermum circinale AWQC310F
Aphanizomenon flos-aquae LD13
Anabaena sp. WA113
Anabaena sp. WA102
Aphanizomenon flos-aquae 2012/KM1/D3
Aphanizomenon flos-aquae NIES-81
Anabaena sp. 90
Anabaena sp. LE011-02
Anabaena sp. AL09
Raphidiopsis brookii D9
Cylindrospermopsis raciborskii MVCC14
Cylindrospermopsis sp. CR12
Cylindrospermopsis raciborskii CS-505
Nostoc sp. PCC 7107
Nostoc sp. MBR 210
Nostoc piscinale CENA21
Anabaena sp. ATCC 33047
Nostoc sp. PCC 7524
Nostoc sp. NIES-3756
Nostoc sp. PCC 7120 (CrhB)
Anabaena variabilis ATCC 29413
Scytonema hofmannii PCC 7110
Tolypothrix bouteillei VB521301
Scytonema tolypothrichoides VB-61278
Mastigocladopsis repens PCC 10914
Scytonema sp. HK-05
Tolypothrix campylonemoides VB511288

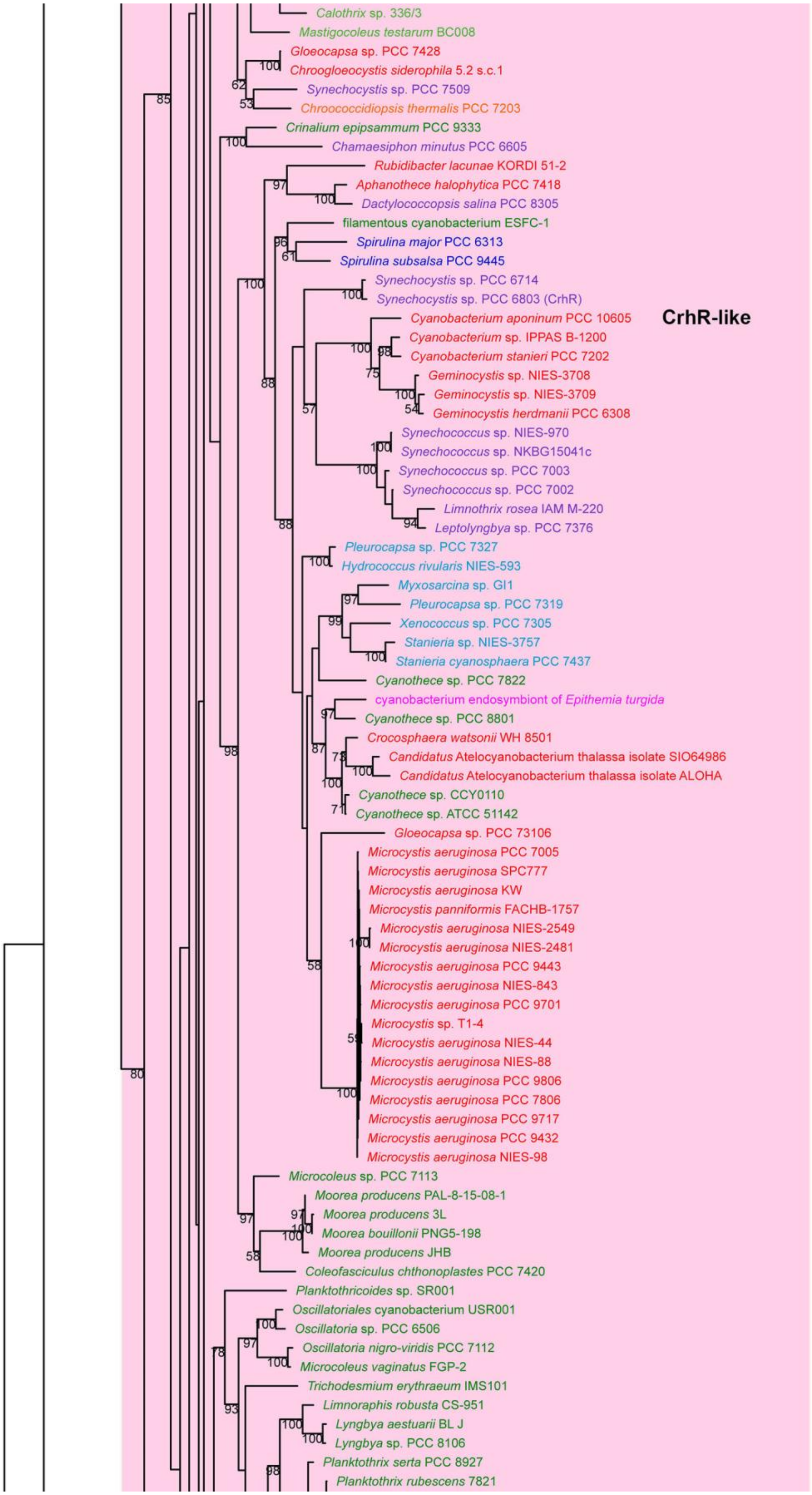
Calothrix sp. 336/3
Mastigocoleus testarum BC008
Gloeocapsa sp. PCC 7428
Chroogloeocystis siderophila 5.2 s.c.1
Synechocystis sp. PCC 7509
Chroococcidiopsis thermalis PCC 7203
Crinalium epipsammum PCC 9333
Chamaesiphon minutus PCC 6605
Rubidibacter lacunae KORDI 51-2
Aphanothece halophytica PCC 7418
Dactylococcopsis salina PCC 8305
filamentous cyanobacterium ESFC-1
Spirulina major PCC 6313
Spirulina subsalsa PCC 9445
Synechocystis sp. PCC 6714
Synechocystis sp. PCC 6803 (CrhR)
CrhR-like
Cyanobacterium aponinum PCC 10605
Cyanobacterium sp. IPPAS B-1200
Cyanobacterium stanieri PCC 7202
Geminocystis sp. NIES-3708
Geminocystis sp. NIES-3709
Geminocystis herdmanii PCC 6308
Synechococcus sp. NIES-970
Synechococcus sp. NKBG15041c
Synechococcus sp. PCC 7003
Synechococcus sp. PCC 7002
Limnothrix rosea IAM M-220
Leptolyngbya sp. PCC 7376
Pleurocapsa sp. PCC 7327
Hydrococcus rivularis NIES-593
Myxosarcina sp. GI1
Pleurocapsa sp. PCC 7319
Xenococcus sp. PCC 7305
Stanieria sp. NIES-3757
Stanieria cyanosphaera PCC 7437
Cyanothece sp. PCC 7822
cyanobacterium endosymbiont of Epithemia turgida
Cyanothece sp. PCC 8801
Crocosphaera watsonii WH 8501
Candidatus Atelocyanobacterium thalassa isolate SIO64986
Candidatus Atelocyanobacterium thalassa isolate ALOHA
Cyanothece sp. CCY0110
Cyanothece sp. ATCC 51142
Gloeocapsa sp. PCC 73106
Microcystis aeruginosa PCC 7005
Microcystis aeruginosa SPC777
Microcystis aeruginosa KW
Microcystis panniformis FACHB-1757
Microcystis aeruginosa NIES-2549
Microcystis aeruginosa NIES-2481
Microcystis aeruginosa PCC 9443
Microcystis aeruginosa NIES-843
Microcystis aeruginosa PCC 9701
Microcystis sp. T1-4
Microcystis aeruginosa NIES-44
Microcystis aeruginosa NIES-88
Microcystis aeruginosa PCC 9806
Microcystis aeruginosa PCC 7806
Microcystis aeruginosa PCC 9717
Microcystis aeruginosa PCC 9432
Microcystis aeruginosa NIES-98
Microcoleus sp. PCC 7113
Moorea producens PAL-8-15-08-1
Moorea producens 3L
Moorea bouillonii PNG5-198
Moorea producens JHB
Coleofasciculus chthonoplastes PCC 7420
Planktothricoides sp. SR001
Oscillatoriales cyanobacterium USR001
Oscillatoria sp. PCC 6506
Oscillatoria nigro-viridis PCC 7112
Microcoleus vaginatus FGP-2
Trichodesmium erythraeum IMS101
Limnoraphis robusta CS-951
Lyngbya aestuarii BL J
Lyngbya sp. PCC 8106
Planktothrix serta PCC 8927
Planktothrix rubescens 7821

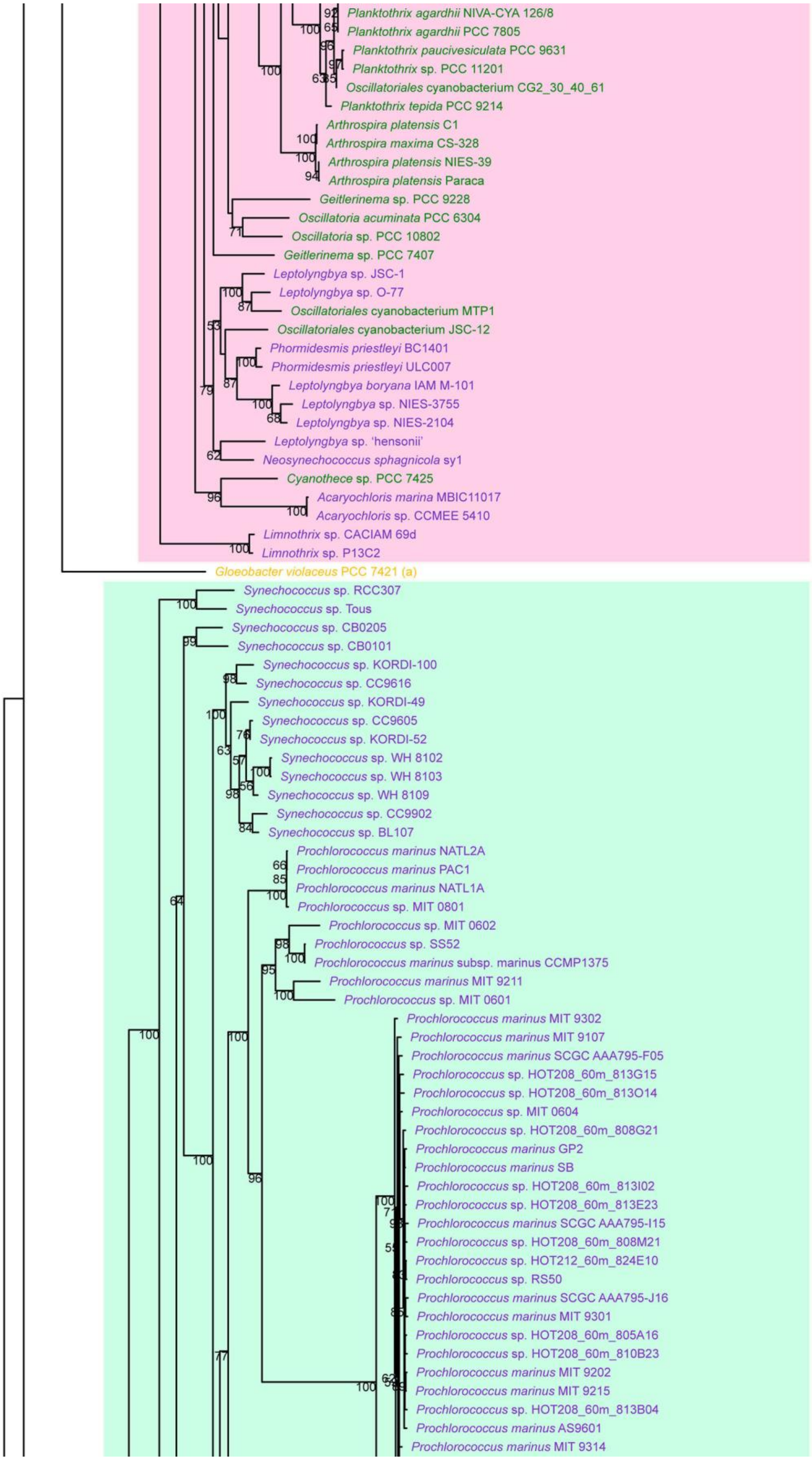

Planktothrix agardhii NIVA-CYA 126/8
Planktothrix agardhii PCC 7805
Planktothrix paucivesiculata PCC 9631
Planktothrix sp. PCC 11201
Oscillatoriales cyanobacterium CG2_30_40_61
Planktothrix tepida PCC 9214
Arthrospira platensis C1
Arthrospira maxima CS-328
Arthrospira platensis NIES-39
Arthrospira platensis Paraca
Geitlerinema sp. PCC 9228
Oscillatoria acuminata PCC 6304
Oscillatoria sp. PCC 10802
Geitlerinema sp. PCC 7407
Leptolyngbya sp. JSC-1
Leptolyngbya sp. O-77
Oscillatoriales cyanobacterium MTP1
Oscillatoriales cyanobacterium JSC-12
Phormidesmis priestleyi BC1401
Phormidesmis priestleyi ULC007
Leptolyngbya boryana IAM M-101
Leptolyngbya sp. NIES-3755
Leptolyngbya sp. NIES-2104
Leptolyngbya sp. 'hensonii'
Neosynechococcus sphagnicola sy1
Cyanothece sp. PCC 7425
Acaryochloris marina MBIC11017
Acaryochloris sp. CCMEE 5410
Limnothrix sp. CACIAM 69d
Limnothrix sp. P13C2
Gloeobacter violaceus PCC 7421 (a)
Synechococcus sp. RCC307
Synechococcus sp. Tous
Synechococcus sp. CB0205
Synechococcus sp. CB0101
Synechococcus sp. KORDI-100
Synechococcus sp. CC9616
Synechococcus sp. KORDI-49
Synechococcus sp. CC9605
Synechococcus sp. KORDI-52
Synechococcus sp. WH 8102
Synechococcus sp. WH 8103
Synechococcus sp. WH 8109
Synechococcus sp. CC9902
Synechococcus sp. BL107
Prochlorococcus marinus NATL2A
Prochlorococcus marinus PAC1
Prochlorococcus marinus NATL1A
Prochlorococcus sp. MIT 0801
Prochlorococcus sp. MIT 0602
Prochlorococcus sp. SS52
Prochlorococcus marinus subsp. marinus CCMP1375
Prochlorococcus marinus MIT 9211
Prochlorococcus sp. MIT 0601
Prochlorococcus marinus MIT 9302
Prochlorococcus marinus MIT 9107
Prochlorococcus marinus SCGC AAA795-F05
Prochlorococcus sp. HOT208_60m_813G15
Prochlorococcus sp. HOT208_60m_813O14
Prochlorococcus sp. MIT 0604
Prochlorococcus sp. HOT208_60m_808G21
Prochlorococcus marinus GP2
Prochlorococcus marinus SB
Prochlorococcus sp. HOT208_60m_813I02
Prochlorococcus sp. HOT208_60m_813E23
Prochlorococcus marinus SCGC AAA795-I15
Prochlorococcus sp. HOT208_60m_808M21
Prochlorococcus sp. HOT212_60m_824E10
Prochlorococcus sp. RS50
Prochlorococcus marinus SCGC AAA795-J16
Prochlorococcus marinus MIT 9301
Prochlorococcus sp. HOT208_60m_805A16
Prochlorococcus sp. HOT208_60m_810B23
Prochlorococcus marinus MIT 9202
Prochlorococcus marinus MIT 9215
Prochlorococcus sp. HOT208_60m_813B04
Prochlorococcus marinus AS9601
Prochlorococcus marinus MIT 9314

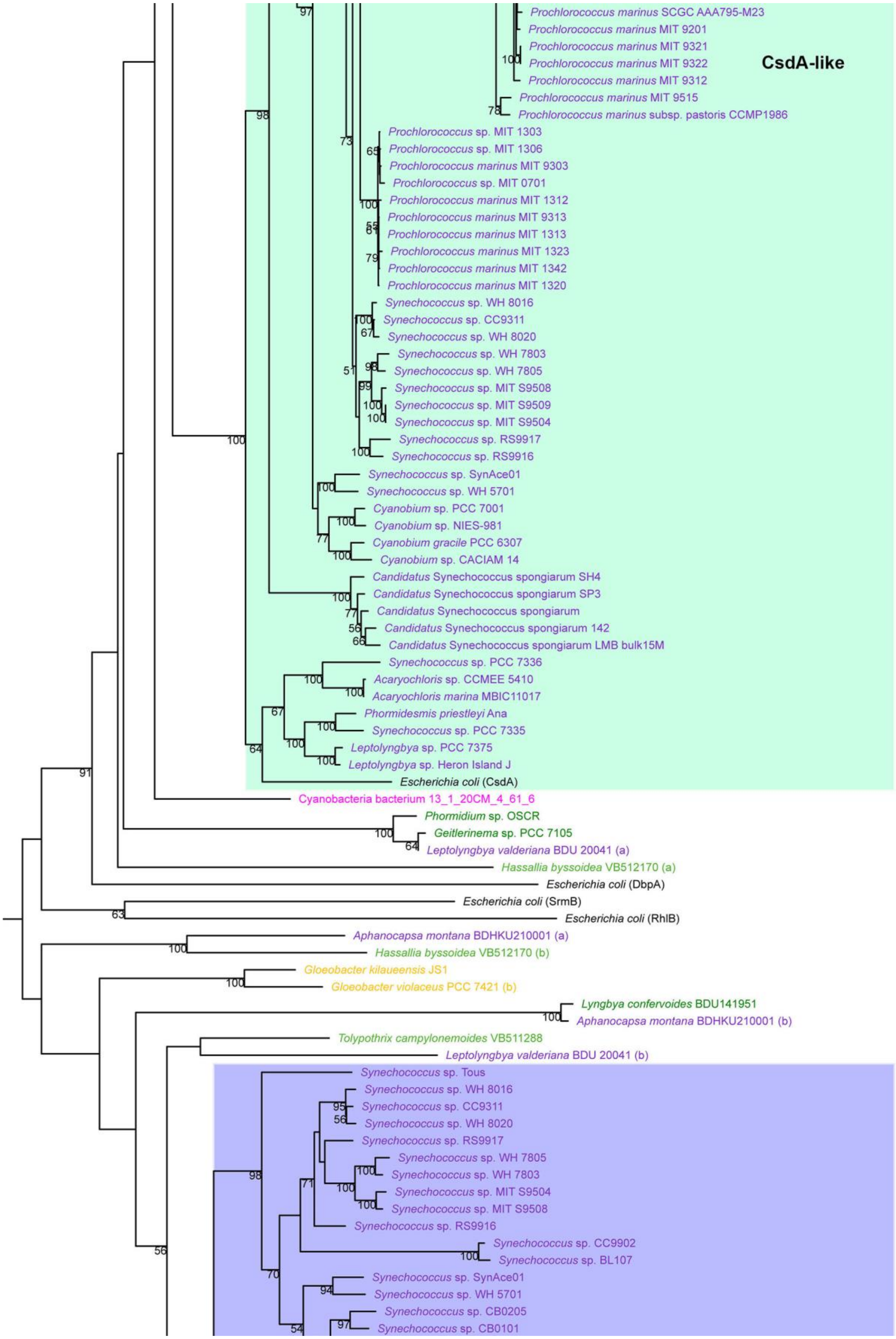
CsdA-like
Prochlorococcus marinus SCGC AAA795-M23
Prochlorococcus marinus MIT 9201
Prochlorococcus marinus MIT 9321
Prochlorococcus marinus MIT 9322
Prochlorococcus marinus MIT 9312
Prochlorococcus marinus MIT 9515
Prochlorococcus marinus subsp. pastoris CCMP1986
Prochlorococcus sp. MIT 1303
Prochlorococcus sp. MIT 1306
Prochlorococcus marinus MIT 9303
Prochlorococcus sp. MIT 0701
Prochlorococcus marinus MIT 1312
Prochlorococcus marinus MIT 9313
Prochlorococcus marinus MIT 1313
Prochlorococcus marinus MIT 1323
Prochlorococcus marinus MIT 1342
Prochlorococcus marinus MIT 1320
Synechococcus sp. WH 8016
Synechococcus sp. CC9311
Synechococcus sp. WH 8020
Synechococcus sp. WH 7803
Synechococcus sp. WH 7805
Synechococcus sp. MIT S9508
Synechococcus sp. MIT S9509
Synechococcus sp. MIT S9504
Synechococcus sp. RS9917
Synechococcus sp. RS9916
Synechococcus sp. SynAce01
Synechococcus sp. WH 5701
Cyanobium sp. PCC 7001
Cyanobium sp. NIES-981
Cyanobium gracile PCC 6307
Cyanobium sp. CACIAM 14
Candidatus Synechococcus spongiarum SH4
Candidatus Synechococcus spongiarum SP3
Candidatus Synechococcus spongiarum
Candidatus Synechococcus spongiarum 142
Candidatus Synechococcus spongiarum LMB bulk15M
Synechococcus sp. PCC 7336
Acaryochloris sp. CCMEE 5410
Acaryochloris marina MBIC11017
Phormidesmis priestleyi Ana
Synechococcus sp. PCC 7335
Leptolyngbya sp. PCC 7375
Leptolyngbya sp. Heron Island J
Escherichia coli (CsdA)
Cyanobacteria bacterium 13_1_20CM_4_61_6
Phormidium sp. OSCR
Geitlerinema sp. PCC 7105
Leptolyngbya valderiana BDU 20041 (a)
Hassallia byssoidea VB512170 (a)
Escherichia coli (DbpA)
Escherichia coli (SrmB)
Escherichia coli (RhlB)
Aphanocapsa montana BDHKU210001 (a)
Hassallia byssoidea VB512170 (b)
Gloeobacter kilaueensis JS1
Gloeobacter violaceus PCC 7421 (b)
Lyngbya confervoides BDU141951
Aphanocapsa montana BDHKU210001 (b)
Tolypothrix campylonemoides VB511288
Leptolyngbya valderiana BDU 20041 (b)
Synechococcus sp. Tous
Synechococcus sp. WH 8016
Synechococcus sp. CC9311
Synechococcus sp. WH 8020
Synechococcus sp. RS9917
Synechococcus sp. WH 7805
Synechococcus sp. WH 7803
Synechococcus sp. MIT S9504
Synechococcus sp. MIT S9508
Synechococcus sp. RS9916
Synechococcus sp. CC9902
Synechococcus sp. BL107
Synechococcus sp. SynAce01
Synechococcus sp. WH 5701
Synechococcus sp. CB0205
Synechococcus sp. CB0101

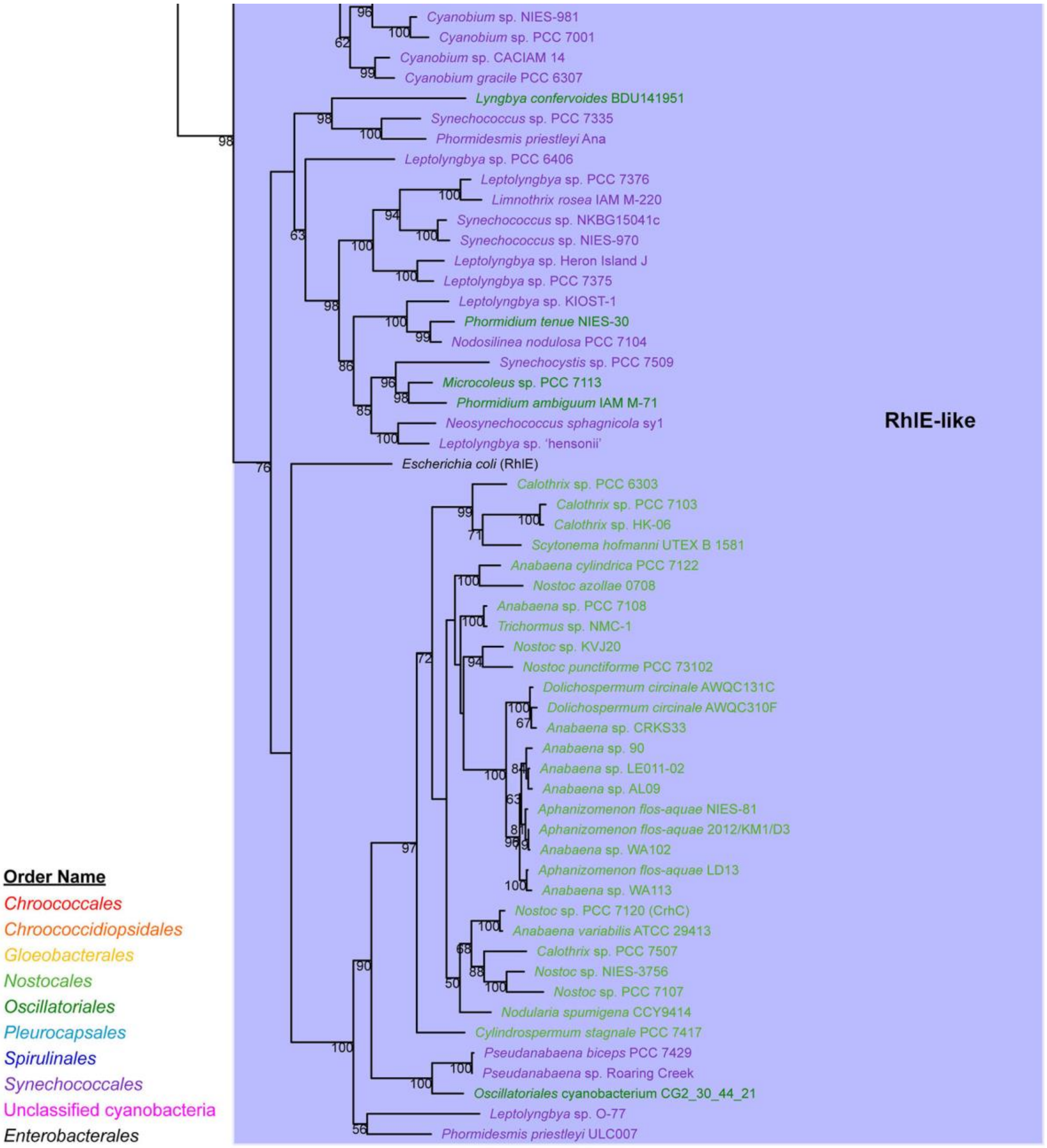

Fig. S2. Maximum likelihood tree of cyanobacterial DEAD-box RNA helicase proteins. The mid-point rooted maximum likelihood tree shows three major clades, corresponding to CrhR-like, CsdA-like and RhlE-like proteins. Highly related strains have been included in this analysis because of the extensive taxonomic issues that exist within cyanobacteria, such as the polyphyletic genus *Synechococcus* [6]. The DEAD-box proteins of *E. coli* (CsdA, DbpA, RhlB, RhlE and SrmB) are included to verify classification of the cyanobacterial proteins. Bootstrap values ≥ 50% shown are at the nodes. Branch lengths are proportional to the mean number of

substitutions per amino acid site as indicated by the scale bar. The strain names are coloured to denote the common taxonomic orders.

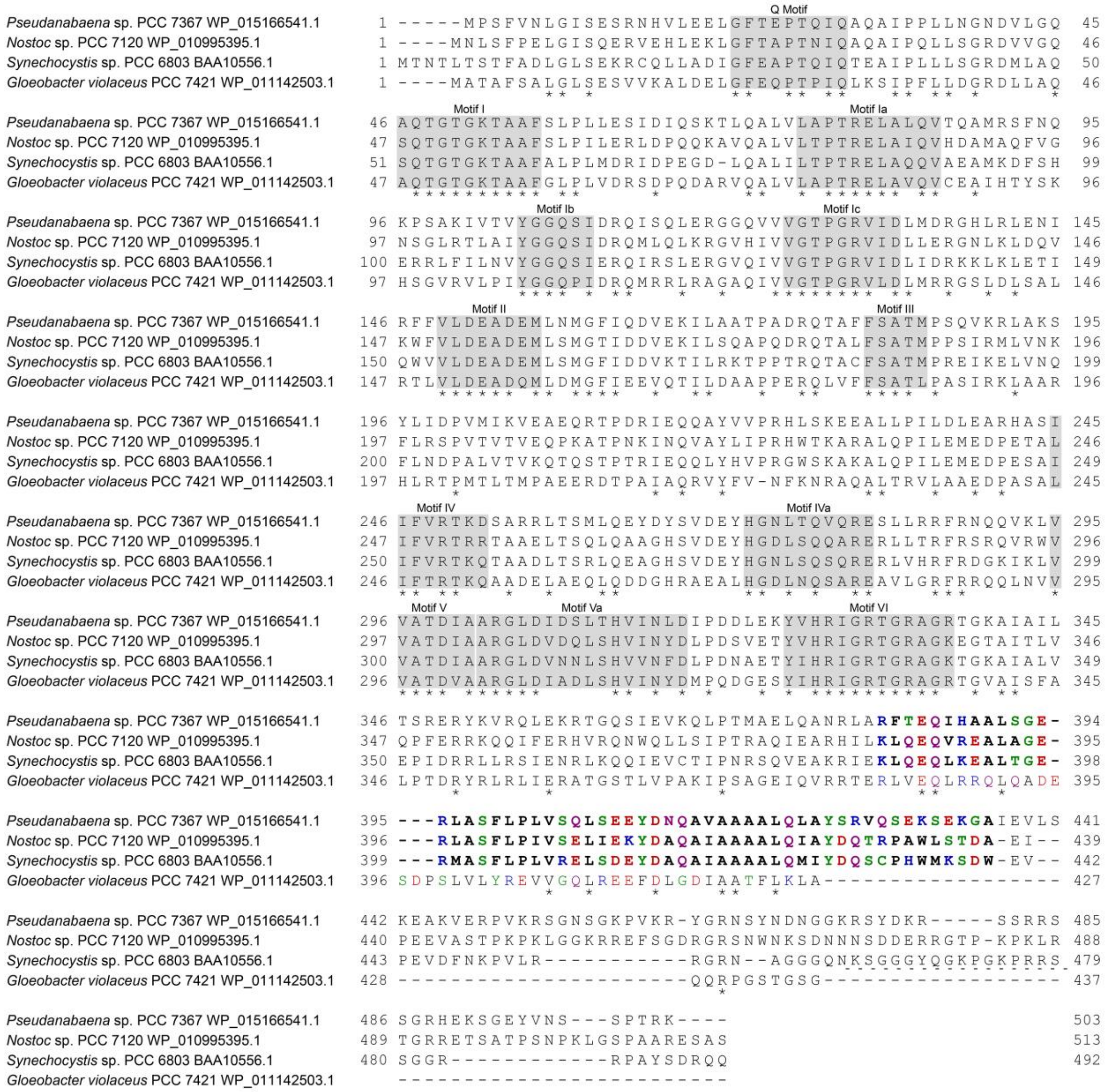

Fig. S3. The *Gloeobacter violaceus* PCC 7421 DEAD-box helicase lacks the CrhR-specific C-terminal extension domain. MUSCLE alignment was performed using selected representatives of the CrhR-like helicase clade against the CrhR-related protein from *G. violaceus*. The twelve conserved DEAD-box RNA helicase motifs are indicated in grey. The conserved sequence motif that defines the cyanobacterial CrhR clade is shown in bold and coloured based on amino acid chemistry.

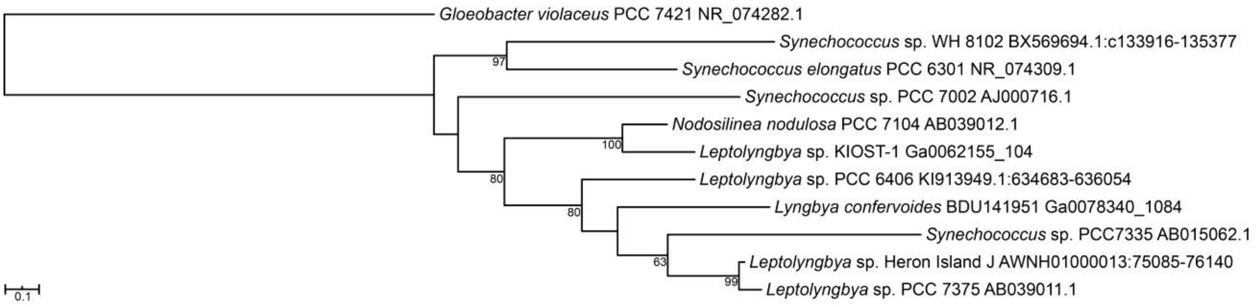

Fig. S4. 16S rDNA maximum-likelihood tree showing the cyanobacterial strains that encode DEAD-box proteins from all three clades of cyanobacterial DEAD-box proteins are closely related. 16S rDNA nucleotide sequences were aligned by MUSCLE and used to construct a maximum-likelihood tree with *Gloeobacter violaceus* PCC 7421 as an outgroup. Several *Leptolyngbya* species and closely related cyanobacteria, as well as several *Synechococcus* species were included to confirm the clustering of the strains that encode all three DEAD-box proteins. Bootstrap values ≥50% are shown at the nodes. Branch lengths are proportional to the mean number of substitutions per nucleotide site as indicated by the scale bar.

References

1. **Linder P**, **Jankowsky E**. From unwinding to clamping – the DEAD box RNA helicase family. *Nat Rev Mol Cell Biol* 2011;12:505–516.

2. **Iost I**, **Dreyfus M**. DEAD-box RNA helicases in *Escherichia coli*. *Nucleic Acids Res* 2006;34:4189–4197.

3. **González-Gutiérrez JA**, **Díaz-Jiménez DF**, **Vargas-Pérez I**, **Guillén-Solís G**, **Stülke J *et al***. The DEAD-box RNA helicases of *Bacillus subtilis* as a model to evaluate genetic compensation among duplicate genes. *Front Microbiol* 2018;9:2261.

4. **de la Cruz J**, **Kressler D**, **Linder P**. Unwinding RNA in *Saccharomyces cerevisiae*: DEAD-box proteins and related families. *Trends Biochem. Sci* 1999;24:192–198.

5. **Umate P**, **Tuteja N**, **Tuteja R**. Genome-wide comprehensive analysis of human helicases. *Commun Integr Biol* 2011;4:118–137.

6. **Robertson BR**, **Tezuka N**, **Watanabe MM**. Phylogenetic analyses of *Synechococcus* strains (cyanobacteria) using sequences of 16S rDNA and part of the phycocyanin operon reveal multiple evolutionary lines and reflect phycobilin content. *Int J Syst Evol Microbiol* 2001;51:861–871.
